# Smelling the romantic partner’s natural body odor increases psychological and autonomic but not cortisol stress responses

**DOI:** 10.1038/s41598-025-27639-w

**Published:** 2026-01-14

**Authors:** Franny B. Spengler, Johannes T. Doerflinger, Josephine A. Noel, Beate Ditzen, Jessica Freiherr, Markus Heinrichs

**Affiliations:** 1 Laboratory for Biological Psychology, Clinical Psychology, and Psychotherapy, Department of Psychology, University of Freiburg, Freiburg, Germany; 2 Institute of Medical Psychology, Heidelberg University Hospital, Bergheimer Str. 20, 69115 Heidelberg, Germany; 3 partner site Mannheim, DZPG (German Center for Mental Health), Heidelberg, Ulm, Germany; 4 Sensory Analytics & Technologies, Fraunhofer Institute for Process Engineering and Packaging IVV, Giggenhauser Straße 35, 85354 Freising, Germany; 5 Department of Psychiatry and Psychotherapy, Friedrich-Alexander-University Erlangen-Nürnberg, Schwabachanlage 6, 91054 Erlangen, Germany; 6 Clinical Biopsychology and Psychotherapy, University of Zurich, Zurich, Switzerland

**Keywords:** Psychology, Human behaviour, Stress and resilience

## Abstract

While social support from romantic partners is known to ameliorate stress responses, it remains unclear whether perceiving a partner’s body odor can elicit similar stress-buffering effects. In this study, 179 participants living in heterosexual romantic relationships underwent either the Trier Social Stress Test (TSST) or a non-stressful control condition while being exposed to their partner’s body odor (collected under standardized conditions over five consecutive nights) or a neutral, non-social control odor presented via an olfactometer. The partner’s odor had no effect on cortisol release. However, contrary to previous findings, subconsciously smelling one’s own partner increased subjective stress and heart rates. Potential underlying mechanisms include the causal misattribution of attraction-related, arousal-induced heart rate increases to the stressful experimental situation, or an evolutionarily adaptive mechanism that amplifies stress responses when a loved one is potentially involved in the threatening situation.

**Supplementary Information:**

The online version contains supplementary material available at 10.1038/s41598-025-27639-w.

## Introduction

Olfactory signals have been shown to crucially modulate social behavior and social interaction^1^. Particularly in romantic interactions and mate selection, human body odors communicate fertility, or genetic compatibility^2^. In addition, humans associate individual body odors with relevant social information^3^. As such, people recognize each other by smell^4^, report to regularly sniff their romantic partner^5^, and experience their partner’s odor as pleasant and comforting^2–7^. Given that the partner’s body odor (from here on ‘partner odor’) could equally well activate calming associations such as a sense of security, or, conversely, sexual arousal^8^, it remains a fascinating yet underexplored question how the subconsciously perceived partner odor might influence subjective and physiological responses in the context of stress.

Supportive social interactions with a partner have been found to buffer against stress^9–12^. Moreover, initial studies suggest that the mere presence of a partner^13^ or even just thinking about them^14^ reduces psychological and physiological stress responses. If the mere presence of a partner is indeed sufficient to yield stress-buffering effects, then their ‘olfactory presence’ (i.e., the romantic partner’s body odor) might also reduce stress, as studies with non-human animals suggest^15, 16^. Initial empirical studies hint at comparable stress-buffering effects in humans. For example, sleeping with a partner’s worn shirt on the pillow has been linked to better sleep quality^17^. Moreover, one study^18^ found that repeatedly smelling a romantic partner’s worn shirt (compared to a stranger’s or an unworn shirt) ameliorated women’s perceived stress before and after a psychosocial stress induction. While partner odor had no effect on cortisol levels overall, cortisol was lowest in a subset of participants who correctly identified the presented odor. In a related study, Granqvist and colleagues^19^ reported reduced discomfort ratings during electric shock application when women smelled their partner’s shirt compared to a shirt worn by themselves. However, discomfort ratings did not differ between smelling the partner’s vs. an unworn shirt. In sum, this emerging research provides initial insights, suggesting stress-buffering effects of the romantic partner’s odor. Yet, although participants in both studies were blinded to the odor condition, actively inhaling the smell of a shirt might elicit beliefs about the odor presented and resemble couple rituals like smelling or wearing the romantic partner’s clothes^7^. It thus remains difficult to disentangle the effects of the belief that one smells his/her partner from the automatic responses induced by subconscious partner odors. To address this issue, we used a computerized olfactometer for odor presentation which permits precise, highly controlled stimulus delivery, allowing us to present chemosensory stimuli continuously and below the threshold of conscious perception, while minimizing fluctuations in delivery or extraneous confounding odors^20^. By doing so, we aimed to create an ecologically valid and highly standardized chemosensory scenario in human research to examine the effects of subconsciously perceived partner odor (compared to a neutral, non-human control odor) in both male and female participants.

While like Hofer et al^18^ we focused on subjective and endocrine stress measures, we additionally analyzed heart rates, as a measure of autonomic nervous system (ANS) activation and arousal. Indeed, contrary to the social support based hypothesis of stress relieving effects of the partner’s odor, it is possible that autonomic responses to the partner’s odor could also be enhanced. As body odor can affect sexual arousal^8^, it is reasonable to assume that physical attraction to one’s partner could lead to arousal-induced heart rate increases when (subconsciously) smelling their body odor^21^. Hence, we assessed subjective ratings on how sexually attractive participants experienced their partner’s odor in general, to capture effects of sexual attraction on heart rate responses. Importantly, effects of sexual attraction to the partner’s odor should be reflected by an increase in heart rate but not in cortisol, as previous studies have found that sexual thoughts do not affect cortisol levels^22^.

Given that prior research suggests that smelling the romantic partner might buffer against stress^18, 19^ and that the olfactory system allows for rapid, automatic processing of socially relevant olfactory cues^4^, we hypothesized that the romantic partner’s odor would reduce stress responses even if presented below the level of conscious awareness (i.e., when the olfactory stimulus is not consciously detected or identified, but could still exert behavioral or physiological effects^23^). We thus employed a 2 × 2 double-blind between subject design, presenting the romantic partner’s body odor (vs. a non-human neutral control odor) via olfactometer to participants, while they were either exposed to psychosocial stress in a standardized social stress task (Trier Social Stress Test, TSST^24)^, or participated in a non-stressful but highly comparable TSST control condition. By testing both partners of heterosexual couples we were further able to explore whether partner odor effects would be observable across both men and women, as sex differences have been reported for olfactory abilities^25^ and the general importance of body odor in romantic relationships^8^. To account for effects of hormonal fluctuations, we only included women that did not use hormonal contraceptives, and conducted the body odor sampling and the experimental testing during the luteal cycle phase. In addition, we assessed subjective ratings on the partner body odor’s attractiveness, which allowed us to exploratorily investigate potential effects of sexual attraction-induced arousal (represented in heart rate increase) as an intriguing aspect of olfactory partner cues.

## Results

### Sample characteristics

Participants were thoroughly prescreened to ensure valuable body odor samples (e.g., not confounded by smoking, taking drugs, or living with pets or small children) and comparable smelling abilities between participants (e.g., by excluding participants taking hormonal contraception, reporting nasal infections or suffering from chronic dysosmia/anosmia; for details on inclusion criteria and procedure see *Methods* section). We collected data in 220 participants (i.e., 110 couples). After exclusions, the final sample for data analyses comprised 179 participants with a mean age of 26.16 years (SD = 3.64, range: 21 to 40) and a mean partnership duration of 39.40 months (SD = 28.73, range: 12 to 175).

### Assessment of stress responsiveness

We calculated linear mixed effects models with a random intercept for each participant to account for the non-independence of measurements (couple ID did not account for variance and models that include random intercepts for couples yield the same results). For all mixed models, we used the lme4 package for R^26^ and estimated degrees of freedom via Satterthwaite’s method implemented in the lmerTest package^27^. The TSST (stress vs. control condition) and odor conditions (partner odor vs. neutral control odor) were dummy coded with the control conditions set at 0.

Analyses predicting perceived stress and cortisol use data from the nine time points (t_0_ to t_8_). We used piecewise linear regression to estimate psychological and endocrine stress responses – a method that fits separate linear trends to different segments of the data, rather than assuming one single trend across the entire period. This can more accurately reflect the typical trajectory of stress responses, which follow a rise-and-fall pattern than a simple linear or polynomial model. Piecewise regression has been previously used in mixed effects models of responses to the TSST^28^. For our models, we divided the time series into two segments: up to the peak of the expected stress response (Time segment 1) and thereafter (Time segment 2). The breakpoints for these segments correspond to established patterns of stress reactivity: the psychological response typically peaks at TSST-task phase (t_2_), whereas cortisol response peaks later, about  20 min after the onset of a stressor (around t_4_). Accordingly, we used t_2_ as the breakpoint for the models predicting psychological responses and t_4_ for models predicting cortisol.  To implement this in our models, we created two time segment variables for each outcome: for Time segment 1, values increase with each time point up to the respective breakpoint (t_2_ for psychological responses, t_4_ for cortisol), and remain constant thereafter; for Time segment 2, values are coded as zero before the breakpoint and increase linearly from the breakpoint onwards. This coding allows the model to estimate separate linear slopes before and after the expected peak. Interactions with Time segment 1 represent the increase in perceived stress and rise of cortisol levels, while interactions with Time segment 2 represent the stress recovery. All models include sex as a covariate.

The three phases (TSST-anticipation, TSST-task, and resting) were entered as predictors into the models predicting heart rates. Phase was coded with two dummy variables with the resting phase as the baseline (*anticipation phase*: TSST-anticipation phase = 1, resting phase = 0, TSST-task phase = 0; *TSST-task phase*: TSST-taskphase = 1, resting phase = 0, TSST-anticipation phase = 0).

Post-hoc ratings revealed that the perceived intensity and pleasantness of the presented odors was comparable between odor conditions, suggesting that the effects found here are not attributable to differences in the chemosensory stimuli’s hedonic value or intensity. Furthermore, participants’ ratings on how stressful they experienced the olfactometer’s nasal cannula during the TSST did not differ between odor conditions, suggesting that results were not confounded by differing degrees of discomfort induced by the olfactory stimulation procedure. The pattern of results across all models remains the same, if we control for trait anxiety, perceived daily stress, and perceived chronic stress (See *Supplemental Materials A*). We also calculated models using the same predictors as reported in the main text with all interaction terms between experimental conditions, time segments (for heart rate data phase), and sex added (for a summary see *Supplemental Materials B*). While main effects of sex are described in *Supplemental Material B*, none of the interactions with sex were significant.

### Partner odor effects on perceived stress

To predict perceived stress, we calculated a linear mixed model with the following predictors: TSST condition, odor condition, Time segment 1, Time segment 2, sex, and interaction terms between each respective time segment and the experimental conditions. All two-way and three-way interaction terms between Time segments, the TSST condition, and the odor condition are included. For a summary of the model see Table 1.

**Table 1:** Mixed linear effects model predicting perceived stress.

Predictors	b		SE(b)	CI	t	df	*p*
Intercept	24.84		2.87	19.20–30.48	8.66	403.83	< 0.001
TSST condition¹	−7.58		3.80	−15.05 – −0.11	−1.99	471.49	0.047
Odor condition²	−5.75		3.84	−13.30–1.80	−1.50	471.93	0.135
Time segment 1³	−1.70		1.21	−4.08–0.68	−1.40	1412.21	0.161
Time segment 2⁴	−1.16		0.38	−1.90 – −0.42	−3.09	1412.72	0.002
Sex⁵	5.24		2.09	1.11–9.38	2.50	174.15	0.013
TSST condition¹ × odor²	15.56		5.43	4.89–26.23	2.87	470.00	0.004
Time segment 1³ × TSST condition¹	7.90		1.72	4.51–11.28	4.58	1412.32	< 0.001
Time segment 1³ × odor²	2.08		1.74	−1.34–5.50	1.19	1412.19	0.233
Time segment 2⁴ × TSST condition¹	−2.46		0.54	−3.51 – −1.40	−4.57	1413.01	< 0.001
Time segment 2⁴ × odor²	0.13		0.54	−0.93–1.19	0.24	1412.58	0.811
Time segment 1³ × TSST condition¹ × odor²	−0.77		2.46	−5.60–4.05	−0.31	1412.25	0.753
Time segment 2⁴ × TSST condition¹ × odor²	−1.59		0.76	−3.09 – −0.10	−2.09	1412.77	0.037
Random effects							
σ^2^	190.81						
τ_00 ID_	172.27						
ICC	0.47						
Observations (*N*_ID_ = 179)	1599						
Marginal R^2^/conditional R^2^	0.145/0.550						

¹ control = 0, stress = 1, ² control = 0, partner = 1, ³ breakpoint at t_2_: t_0_ = 0, t_1_ = 1, t_2_ to t_8_ = 2, ⁴ breakpoint at t_2_: t_0_ to t_2_ = 0, t_3_ = 1, t_4_ = 2, t_5_ = 3, t_6_ = 4, t_7_ = 5, t_8_ = 6, ⁵ male = 0, female = 1

**Fig. 1:**
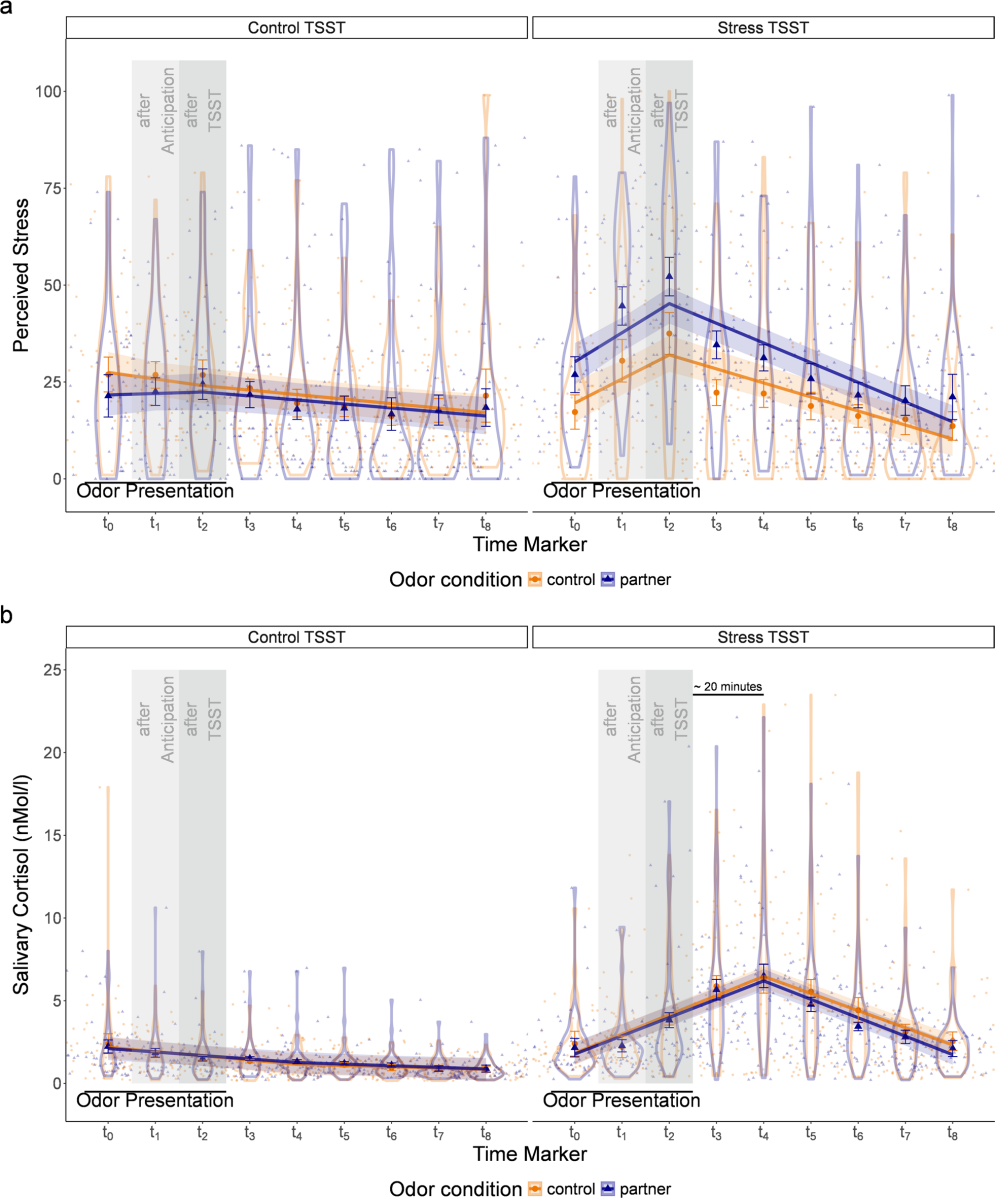
Perceived stress (**a**) and salivary cortisol (**b**) by experimental conditions.Transparent dots represent raw data points. Solid points show mean values for each measurement. Error bars show 95%-confidence intervals of the mean based on random intercepts at the participant level. Violins visualize distributions. Solid lines show the prediction of the mixed model with 95% confidence intervals as shaded areas.

Significant main effects were observed for sex (indicating higher stress levels for women), the TSST condition, and Time segment 2. Both the two-way interaction between the TSST condition and Time Segment 1 as well as between the TSST condition and Time segment 2 were significant, reflecting that stress increased until t_2_ (i.e., the end of the TSST-task phase) and decreased from then on for participants in the TSST stress condition (but not in the control condition). The two-way interaction between the TSST and odor conditions was significant (suggesting more pronounced odor effects in the TSST stress than the control condition). These effects were qualified by a significant three-way interaction between TSST and odor conditions with Time segment 2. This interaction pattern can be interpreted as follows: beginning at t_0_ perceived stress in the TSST stress condition was higher in the partner odor condition compared to the control odor condition, but beginning from t_2_ (peak stress) this difference decreased over time. For a visualization of the findings see Fig. 1a.

### Partner odor effects on cortisol response

To predict the cortisol response, we calculated a linear mixed model with the same predictors as used for perceived stress (time segments corresponding to the expected cortisol pattern). For a summary of the results see Table 2. Significant effects were observed for Time segment 1, sex, and the interactions of the TSST-condition with both time segments respectively.

For a visualization of the results, see Fig. 1b. In the TSST stress condition, cortisol levels increased with a delay reaching their peak at around 20 min after the end of the TSST (t_4_) and decreased thereafter reaching similar levels to the first measurement at the end of the experiment. This typical cortisol response was unaffected by odor. The sex main effect is due to lower cortisol levels in women.

**Table 2:** Mixed linear effects model predicting cortisol levels.

Predictors	b		SE(b)	CI	t	df	*p*
Intercept	2.56		0.37	1.82–3.29	6.87	248.83	< 0.001
TSST condition¹	−0.40		0.48	−1.35–0.55	−0.83	269.86	0.407
Odor condition²	−0.08		0.49	−1.04–0.87	−0.17	270.01	0.862
Time segment 1³	−0.26		0.06	−0.38 – −0.14	−4.12	1422.98	< 0.001
Time segment 2⁴	−0.07		0.06	−0.20–0.05	−1.17	1422.98	0.244
Sex⁵	−0.74		0.31	−1.35 – −0.13	−2.39	173.98	0.018
TSST condition¹ × odor²	0.14		0.69	−1.21–1.50	0.21	269.29	0.834
Time segment 1³ × TSST condition¹	1.42		0.09	1.24–1.60	15.88	1422.98	< 0.001
Time segment 1³ × odor²	0.05		0.09	−0.13–0.23	0.53	1422.98	0.599
Time segment 2⁴ × TSST condition¹	−0.96		0.09	−1.14 – −0.79	−10.75	1423.08	< 0.001
Time segment 2⁴ × odor²	−0.02		0.09	−0.20–0.15	−0.27	1422.98	0.787
Time segment 1³ × TSST condition¹ × odor²	−0.10		0.13	−0.35–0.15	−0.81	1422.98	0.419
Time segment 2⁴ × TSST condition¹ × odor²	−0.05		0.13	−0.30–0.20	−0.42	1423.03	0.678
Random effects							
σ^2^	2.25						
τ_00 ID_	3.96						
ICC	0.64						
Observations (*N*_ID_ = 179)	1610						
Marginal R^2^/conditional R^2^	0.317/0.753						

¹ control = 0, stress = 1, ² control = 0, partner = 1, ³ breakpoint at t_4_: t_0_ = 0, t_1_ = 1, t_2_ = 2, t_3_ = 3, t_4_ to t_8_ = 4, ⁴ breakpoint at t_4_: t_0_ to t_4_ = 0, t_5_ = 1, t_6_ = 2, t_7_ = 3, t_8_ = 4, ⁵ male = 0, female = 1

### Partner odor effects on heart rate

We calculated a mixed linear model comparing odor and TSST effects across the three relevant phases of the experiment: the TSST-anticipation phase, TSST-task phase, and a resting phase at the end of the experiment. The phases were dummy coded with two variables representing the TSST-anticipation and TSST-task phase respectively with the resting phase set as the reference category. The model had the following main effect terms: TSST condition, odor condition, the phase dummy variables, sex, and trait anxiety. Due to the dummy coding of the phase variables, all phase-related effects are relative to the resting phase. As interaction effects, all two-way and three-way interactions between phase and the experimental conditions were included. For a summary of the model, see Table 3. Significant main effects were observed for the two phase variables, and sex. In addition, the two-way interaction effects of both the TSST-condition variable and the odor condition variable with the phase variables were significant. These effects were qualified by a significant three-way interaction between the TSST phase variable, the TSST condition and the odor condition. The significant effect of sex indicates that women had higher heart rates than men.

**Fig. 2:**
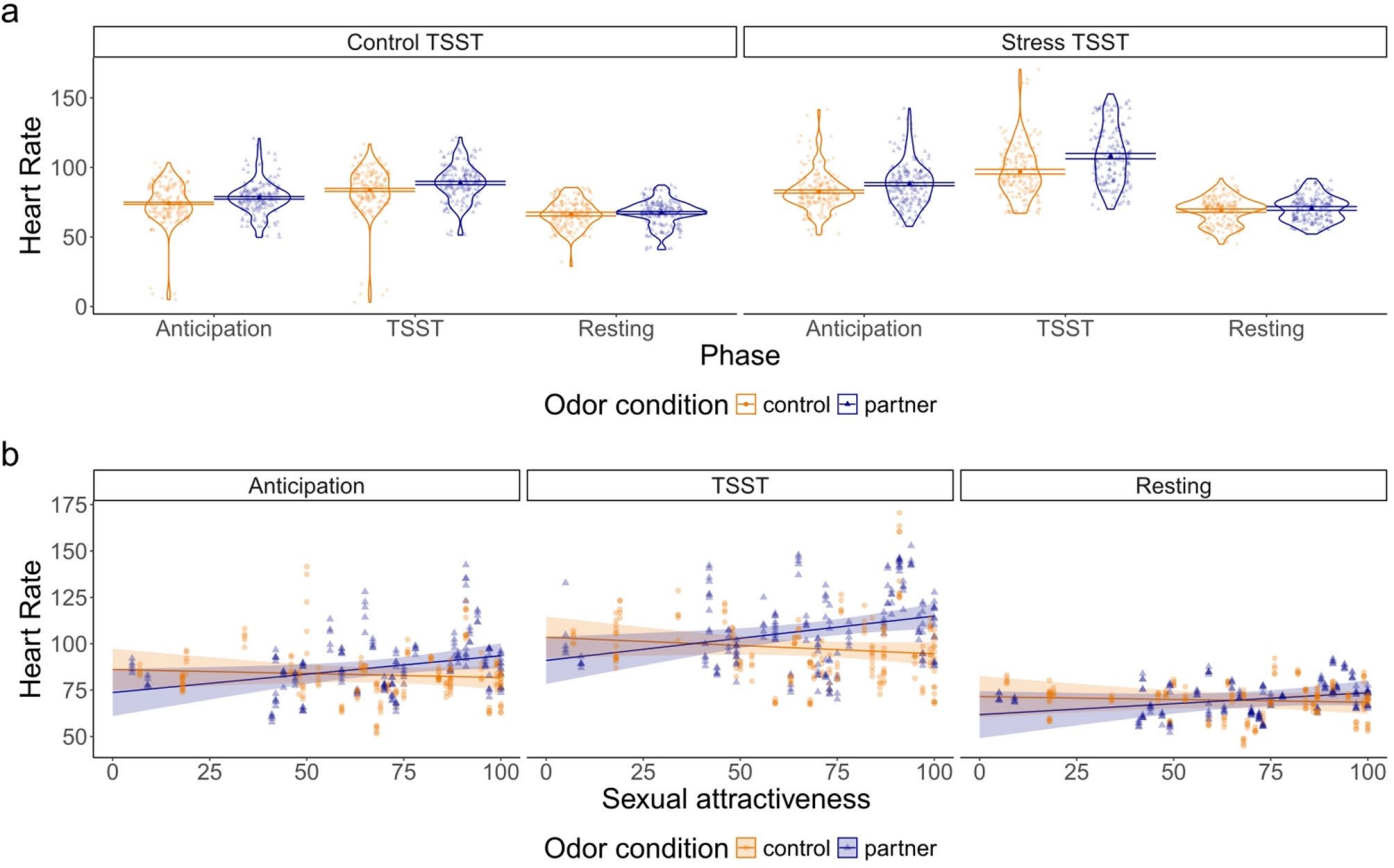
Heart rates in beats per minute experimental conditions (**a**) and Heart rates in the TSST stress condition by phase and general partner odor sexual attractiveness (**b**). Odor was presented during the TSST-anticipation- and the TSST-task phase. Dots represent raw data points. (**a**) Mean values are depicted for each measurement. Violins visualize distributions. Error bars show 95%-confidence interval. (**b**) Solid lines show the prediction of the mixed model with shaded areas for the 95%-confidence interval.

The pattern of results is visualized in Fig. 2a. As can be seen, heart rate was lowest during the resting phase, higher during the TSST-anticipation phase and highest during the TSST-task phase. Additionally, heart rate was higher, when a partner’s odor was present, compared to the neutral odor condition. This difference was more pronounced in the TSST stress condition than in the TSST control condition. The biggest effect of odor on heart rate was observed in the TSST stress condition during the TSST-task phase.

We observed significant positive correlations between perceived stress, heart rate and cortisol release at all corresponding time points (with an expected time gap of 20 min for cortisol release; for details on single correlations see *Supplemental Materials C*).

**Table 3:** Mixed linear effects model predicting heart rates.

Predictors	b	SE(b)	CI	t	df	*p*
Intercept	62.83	2.27	58.34–67.32	27.65	151.74	< 0.001
TSST condition¹	3.33	2.82	−2.25–8.90	1.18	155.16	0.240
Odor condition²	1.25	2.89	−4.46–6.97	0.43	155.27	0.665
TSST-anticipation phase³	7.76	0.75	6.30–9.23	10.39	2036.00	< 0.001
TSST-task phase⁴	17.40	0.75	15.94–18.87	23.28	2036.00	< 0.001
Sex⁵	6.54	1.99	2.59–10.48	3.28	141.00	0.001
TSST condition¹ × odor condition²	−0.75	4.08	−8.82–7.31	−0.18	155.12	0.854
TSST condition¹ × TSST-anticipation phase³	5.97	1.06	3.89–8.04	5.64	2036.00	< 0.001
TSST condition¹ × TSST-task phase⁴	10.62	1.06	8.55–12.70	10.05	2036.00	< 0.001
Odor condition² × TSST-anticipation phase³	3.01	1.09	0.87–5.14	2.76	2036.00	0.006
Odor condition² × TSST-task phase⁴	4.02	1.09	1.89–6.16	3.70	2036.00	< 0.001
TSST condition¹ × odor condition² × TSST-anticipation phase³	0.76	1.53	−2.23–3.76	0.50	2036.00	0.617
TSST condition¹ × odor condition² × TSST-task phase⁴	5.60	1.53	2.61–8.60	3.67	2036.00	< 0.001
Random effects						
σ^2^	53.11					
τ_00 ID_	139.37					
ICC	0.72					
Observations (*N*_ID_ = 146)	2190					
Marginal R^2^/conditional R^2^	0.457/0.850					

¹ control = 0, stress = 1, ² control = 0, partner = 1, ³ dummy coded, TSST-anticipation phase = 1, resting phase = 0, TSST-task phase = 0, ⁴ dummy coded, TSST-task phase = 1, TSST-anticipation phase = 0, resting phase = 0, ⁵ male = 0, female = 1

#### Exploring the role of sexual attraction

To explore whether sexual attraction contributes to the higher heart rate observed when partner odor was presented, we added the participants’ rating of how ‘sexy’ their partner smelled (variable: sexual attractiveness) as an additional moderator (including all two-, three, and four-way interactions between sexual attractiveness, odor condition, TSST condition, and phase). This model included a significant four-way interaction between the TSST condition, odor, the phase variables, and sexual attractiveness (TSST-anticipation phase: *b* = 3.44, *t*(2028.0) = 2.18, *p* =.030; TSST-task phase: *b* = 8.68, *t*(2028.0) = 5.50, *p* <.001). The full model is summarized in *Supplemental Materials D.* As effects of the partner odor’s sexual attractiveness were more pronounced in the TSST stress condition, we subsequently ran a mixed model that only included participants in the TSST stress condition with heart rate data available (*N* = 74) to decompose the observed four-way interaction effect. In this model the predictors were: odor condition, phase (both dummy variables), sexual attractiveness, and all interaction terms. The model is summarized in Table 4. We observe significant main effects and two-way interactions of sex and the TSST-anticipation- and TSST-task phase variables. These were qualified by a significant interaction of odor condition, TSST phase, and sexual attractiveness. The model predictions are visualized in Fig. 2b.

As can be seen, in the two phases where odor stimuli were present (i.e., the TSST-anticipation and the TSST-task phase), the partner odor’s sexual attractiveness was positively related to heart rate. This trend was more pronounced in the TSST-task phase. Only for those participants who rated their partner’s odor as highly sexually attractive did the confidence intervals of the two odor conditions not overlap – in other words the effect of the presented odor on heart rate was conditional to the participants’ sexual attraction such that odor significantly predicted heart rate only when the partner odor’s sexual attractiveness was rated as high.

To test the robustness of these findings we repeated the same analysis, but instead of sexual attractiveness ratings of partner odor, we used three different indicators related to sexual attraction as moderators to investigate the interplay between odor stimuli and sexual attraction-induced arousal affecting heart rates: *first*, participants’ frequency of sexual intercourse – assuming that those participants who often engage in sexual intercourse with their partner also associate their partner’s odor more with sexual arousal; *second*, participants’ rating of general loveliness of their partner’s odor; *third* participants ratings of how pleasant the presented odor stimulus was. These analyses are summarized in *Supplemental Materials E* and the overall pattern of results provides converging evidence with findings reported here.

**Table 4:** Mixed linear effects model predicting heart rates with partner odor sexual attractiveness as a moderator.

Predictors	b	SE(b)	CI	t	df	*p*
Intercept	66.26	2.40	61.49–71.03	27.65	75.15	< 0.001
Odor condition¹	0.47	2.99	−5.48–6.42	0.16	77.30	0.875
TSST-anticipation phase²	13.72	0.85	12.06–15.39	16.16	1028.00	< 0.001
TSST-task phase³	27.98	0.85	26.32–29.65	32.96	1028.00	< 0.001
Sexual attractiveness ⁴	−0.76	1.97	−4.69–3.17	−0.39	77.50	0.700
Sex⁵	6.25	2.91	0.45–12.05	2.15	69.00	0.035
Odor condition¹ × TSST-anticipation phase²	3.71	1.22	1.32–6.10	3.05	1028.00	0.002
Odor condition¹ × TSST-task phase³	9.57	1.22	7.18–11.96	7.86	1028.00	< 0.001
Odor condition¹ × sexual attractiveness ⁴	3.74	2.98	−2.18–9.67	1.26	77.51	0.212
Sexual attractiveness ⁴ × TSST-anticipation phase²	−0.34	0.81	−1.94–1.25	−0.42	1028.00	0.674
Sexual attractiveness ⁴ × TSST-task phase³	−1.49	0.81	−3.08–0.11	−1.83	1028.00	0.068
Odor condition¹ × TSST-anticipation phase² × sexual attractiveness ⁴	2.38	1.23	−0.03–4.79	1.94	1028.00	0.052
Odor condition¹ × TSST-task phase³ × sexual attractiveness ⁴	4.56	1.23	2.15–6.97	3.72	1028.00	< 0.001
Random effects						
σ^2^	68.43					
τ_00 ID_	148.20					
ICC	0.68					
Observations (*N*_ID_ = 74)	1110					
Marginal R^2^/conditional R^2^	0.496/0.840					

¹ control = 0, partner = 1, ² dummy coded, TSST-anticipation phase = 1, resting phase = 0, TSST-task phase = 0, ³ dummy coded, TSST-task phase = 1, TSST-anticipation phase = 0, resting phase = 0, ⁴ z-standardized, ⁵ male = 0, female = 1

## Discussion

In humans – alike other animals - olfactory signals significantly influence social communication and interaction. However, while there is evidence that odors can serve as social cues and thereby modulate affect and activate memories, there is still little data on how the romantic partner’s scent might influence psychobiological stress experiences. We hypothesized that subconsciously smelling the partner’s body odor should evoke perceptions of social safety and support and buffer against psychosocial stress. Yet, contrary to previous findings, partner odor increased perceived stress and heart rate, whereas cortisol release was unaffected by odor presentation. Further analyses revealed greater cardiovascular partner odor effects in participants who rated their partner’s odor as sexually attractive, suggesting that the observed heart rate increases may also represent subconsciously triggered sexual arousal.

At a first glance, the observed partner odor-induced increase in perceived stress and heart rates seems to contradict previous research on stress buffering effects of social support and olfactory partner cues^11, 29^. However, several studies highlight the complexity of social support mechanisms, suggesting that the perceived quality and type of social support critically influence its effectiveness (e.g.,^9, 11, 30^). Subconscious partner cues (like body odor) could possibly be insufficient to mirror the support quality of consciously perceived partner support and hence fail to elicit stress relief.

Findings on partner odor effects on stress are very limited and still rather inconclusive: One study reported stress-ameliorating effects when women smelled their partners^18^, while another found no differences in discomfort ratings between partner and neutral odor presentation under physiological stress^19^. A potential explanation for our contradictory findings of increased heart rates and subjective stress for partner odor might lie in methodological differences in in the presentation of the chemosensory stimuli. Previous studies involved explicit partner odor exposure, such as actively inhaling a shirt, whereas our study presented partner odors subconsciously and within a continuous air flow via an olfactometer, to enable maximally standardized odor presentation. Consequently, the stress-buffering effects in Hofer and colleagues’ study^18^ might be attributable to evoked beliefs about the odor presented (i.e., human odor) and conscious associations one might have with their romantic partner. Our study, however, allows to draw consequences on automatic effects of the *subconsciously *perceived chemosensory cue. In sum, our findings suggest that the mere subconscious chemosensory partner cue might not be sufficient to mirror support effects of an actually present partner, and to induce a sense of security and calm. Rather, conscious perception of the partner’s (chemosensory) presence, accompanied by positive associations, appears essential for achieving stress-buffering effects, as seen with partner-related cues in other modalities (e.g., presence, thoughts, visual cues^14).^ While we found no sex differences in partner odor effects, further research could contribute to a more nuanced understanding of the role of sex-specific social support perceptions and odor sensitivity, and the impact of age and couple-dependent arousal evaluations.

Notably, we found partner odor effects on subjectively reported stress and arousal-related autonomic nervous system responses (i.e., heart rate), while partner odor did not affect hypothalamic–pituitary–adrenal axis activation (i.e., cortisol responsiveness). This aligns with previous research suggesting that the mere presentation of social cues may not be sufficient, but rather may interact with a challenging situation, such as social stress, to amplify response patterns. Importantly, inconsistent responses across these three stress measures (subjective stress, heart rates, cortisol release) are common in studies on both social support effects and partner odor effects (e.g^11, 18^.,) and it remains an open question, under which conditions physiological and psychological stress responses align or dissociate^31^.

Overall, our data reveal aggravated stress responses when exposed to the romantic partner’s body odor in situations of psychosocial stress, suggesting arousing, rather than calming effects of subconscious olfactory partner cues. We consider three possible explanations: First, the partner’s odor might have had detrimental effects in that it intensified aversive stress experiences. Previous research indicates that chemosensory signals of stress can be transmitted between individuals, evoking physiological or emotional responses in recipients^32^. Future studies should further examine the potential for stress contagion effects via partner odor under varying contexts and conditions. Moreover, Granqvist and colleagues^19^ found partner odor induced increases in physiological arousal (i.e., skin conductance) during stress within a subgroup of insecurely attached participants. In the present study, however, participants were prescreened for stable, high-quality partnerships, thus minimizing the expected number of participants for whom the partner’s odor could have carried mainly stressful associations. Second, the associations between heart rate increase and the odor’s perceived sexual attractiveness suggests that the heart rate effects found here may reflect arousal rather than cardiovascular stress. The increase in *perceived* stress potentially arises from causal misattribution of the subconsciously triggered arousal on the experimental context^33, 34^ or by excitation transfer^35^. Finally, the partner odor-induced increase in perceived stress and heart rate might also represent an evolutionarily adaptive reaction. This reaction could boost an individual’s energy and focus^36^ to optimize performance under stress when a loved person is involved in the potentially threatening situation, to ensure mutual well-being. Additionally, the chemosensory partner cue may also evoke motives to impress the (presumably near-by) loved one by demonstrating optimal performance (i.e., high fitness), again aligning with stronger effects in those rating their partner’s odor as sexually attractive.

### Limitations and future research

Our study is the first to examine the effect of standardized, subconscious partner odor presentation (via a computerized olfactometer) on subjective, endocrine and cardiovascular stress markers in both male and female participants. Moreover, our study design excels in that it applies maximally standardized body odors and includes a highly controlled non-stressful control condition. Yet, due to rigorously controlled conditions, we were limited in the amount of factors included in the study design. Future research should explicitly compare responses to partner odors versus other familiar or unfamiliar social odors to definitively confirm that the effects are partner odor-specific. However, the positive correlations with subjective ratings of the partner odor’s attractiveness and loveliness strongly suggest that the here observed effects are tied to the odor of a romantic partner. Moreover, we collected data in relatively young, heterosexual, monogamous couples. Future research exploring whether our findings generalize to diverse relationship forms or older age groups, which may experience hormonal changes that do not follow the typical fluctuation pattern of the menstrual cycle and might differentially affect body odor and olfactory ability, would be highly valuable. To elucidate the potential underlying mechanisms, future studies could, for example, examine partner odor effects on stress task performance, use implicit measures of sexual arousal, or have a deeper look into different facets of partnership quality, potentially moderating stress buffering partner support effects. Lastly, while our methodological approach of continuous stimulation aimed to maximize ecological validity by most closely resembling a partner’s actual presence, future studies employing different forms of olfactory stimulation, such as intermittent or burst stimulation, would be highly valuable to better understand potential impact of sensory habituation in partner odors.

## Conclusion

In contrast to initial pilot studies, we found no evidence for stress buffering effects of the romantic partner’s body odor. Instead, subconscious exposure to the romantic partner’s odor increased subjective stress and heart rate responses to psychosocial stress. These effects could either be explained by misattribution of sexual attraction-induced arousal, or by evolutionarily adaptive increases in stress responsiveness that enable optimal performance when the partner is involved in a stressful situation. Taken together, our results shed light on the stress-modulating effects of subconsciously presented olfactory partner cues, underlining the complex role of chemosensory signaling in social interaction.

## Method

### Study design

To examine the effect of the romantic partner’s body odor on stress reactivity in a standardized social stress test (Trier Social Stress Test, TSST^24^), this study followed a randomized, double-blind, 2 (TSST: stress vs. control) by 2 (odor: partner vs. control) between-participants design. Both partners within a couple were allocated to the same experimental condition. Outcome measures comprised self-rated perceived stress (as a measure of subjective stress), heart rates (assessing stress- and arousal-related ANS activation), and salivary cortisol (assessing endocrine stress response). The study’s design and implementation were approved by the ethics committee of the University of Freiburg (ethics vote #547/15). All of the procedures in the present study were carried out in accordance with the institutional and national ethical guidelines for human studies, and the guidelines proposed in the Declaration of Helsinki. All participants gave written informed consent for their study participation.

### Participants

Based on the result of a power analysis, we aimed for a sample size of *N* = 204 (i.e., 102 couples) in order to be able to detect medium sized effects (*f* = 0.25) with a power of 1-*β* = 80%. We calculated initial power based on a standard ANOVA, as power analysis for mixed models requires additional assumptions regarding random effect variances and intra-class correlations that are generally not available prior to data collection. Post-hoc sensitivity analyses using actual sample sizes and standard ANOVA models were performed to estimate detectable effect sizes; we note that these provide a conservative approximation of statistical power within our mixed model framework. As we expected approximately 5% data loss within the final sample, we recruited 220 participants (110 couples). We collected data from both partners of heterosexual couples that were in a monogamous relationship for at least one year and reported good partnership quality (indicated via a total score of ≥ 50 in the Partnership Questionnaire^37)^.

We included only healthy, normosmic participants between 22 and 40 years that were not suffering from a chronic or neurological disorders and were not taking any psychoactive medication, nor consuming alcohol (more than 60 g a day) or drugs. As both olfaction and stress responsiveness are known to depend on hormonal fluctuations^25, 38^, women were only included if they reported to have a regular menstrual cycle and were not pregnant, breast feeding, or taking hormonal contraceptives. To avoid alterations of the natural body odor, participants were excluded if they reported to live with children below the age of 10 or with pets with fur or feathers, or if they were smoking on a regular basis (more than once a week). Moreover, at the time point of testing both partners had to be free of any viral upper respiratory tract infections or acute allergic reactions including pollinosis. In order to avoid factors that might confound stress reactivity in the TSST participants were additionally excluded if they had a body mass index below 18.5 or above 30, if they were taking medication containing cortisone or lithium, if they had been in psychotherapeutic or psychiatric treatment in the past six months, if they worked at night or in shifts, they studied psychology, or had previously participated in the TSST.

Out of the 220 recruited participants (i.e., 110 couples) who met all inclusion criteria, 41 participants were excluded from data analysis for the following reasons: intake of illicit medication, drugs or substantial amounts of alcohol prior to study participation (*n* = 7), olfactory impairments or smelling problems during the experiment (*n* = 15), language issues (*n* = 2), technical malfunctions and procedure irregularities (*n* = 15). Moreover, we excluded one participant who consciously perceived the presented odor and another participant with cortisol levels (range: 21.51 nmol/l to 71.57 nmol/l) well outside the expected range^39^.

After exclusion, the final sample was *N =* 179 (91 women, 88 men, from 108 couples; in some couples only data from one partner but not the other was included), with a mean age of 26.24 years (*SD* = 3.67, range: 21 to 40) and a mean partnership duration of 39.40 months (*SD* = 28.73, range: 12 to 175). A sensitivity analysis indicated that with this sample size in an ANOVA the minimum effect size that can be detected at a power of 1-β = 80% is f = 0.25. Because not all participants provided data for all variables, sample sizes differ between analyses. Due to equipment failure, heart rate data for 33 participants could not be recorded resulting in an effective sample size of 146 for heart rate analyses. A sensitivity analysis indicated that with this sample size in an ANOVA the minimum effect size that can be detected at a power of 1-β = 80% is f = 0.28. The amount of data-loss did not differ significantly between experimental conditions, |*z*s| < 0.91, *p*s > 0.36. Data were collected between March 2016 and October 2021; it was paused for seven months when COVID-19 restrictions were in place. For a summary of descriptive statistics and sample characteristics, see Table 5.

**Table 5:** Sample characteristics.

Characteristic	N	Entire sample	Control odor, TSST control	Control odor, TSST stress	Partner odor, TSST control	Partner odor, TSST stress
		179	46^*1*^	45^*1*^	43^*1*^	45^*1*^
Age	179	26.2 (3.7)	25.6 (3.3)	27.8 (4.2)	26.1 (3.3)	25.5 (3.5)
Sex²	179	91 (51%)	23 (50%)	20 (44%)	21 (49%)	27 (60%)
Marital status³	177	13 (7.3%)	1 (2.2%)	5 (11%)	5 (12%)	2 (4.5%)
(Missing)		2	1	0	0	1
Duration of partnership⁴	179	39.4 (28.7)	36.4 (29.8)	48.3 (37.2)	40.3 (23.2)	32.7 (19.9)
Smoking⁵	178	17 (9.6%)	9 (20%)	3 (6.7%)	4 (9.3%)	1 (2.2%)
(Missing)		1	1	0	0	0
Odor identification⁶	175	13.1 (1.4)	13.0 (1.5)	13.3 (1.3)	12.9 (1.4)	13.2 (1.5)
(Missing)		4	1	1	2	0
Odor threshold⁶	174	9.8 (5.0)	10.9 (8.7)	9.7 (2.6)	9.0 (2.2)	9.2 (2.9)
(Missing)		5	1	1	3	0
Smelling ability⁷	179	66.6 (20.8)	62.7 (20.8)	68.1 (22.0)	68.5 (21.0)	67.4 (19.3)

¹ *Mean* (*SD*); *n* (%), ² women in the sample, ³ married participants, ⁴ in months, ⁵ 1 = smoking (although regular smokers were excluded from the study, occasional smokers, who smoked less than once a week are included), ⁶ values from the Sniffin’ Sticks Test, ⁷ self-rated (from 0 to 100)

### Procedure

Participants were recruited via public announcements. If both partners met all inclusion criteria in an online screening questionnaire, they were contacted via phone to double-check eligibility and to provide them with relevant information on the study’s procedures. Those proving eligible were then invited for a first individual appointment, where they were tested for sufficient olfactory abilities using the odor identification task and the odor threshold task of the ‘Sniffin’ Sticks’ battery^40^. In line with the existing normative data^41^, couples were excluded from further testing if one or both partners reached an odor threshold below 6 and identified less than 12 out of 16 odors correctly. At the end of the appointment, normosmic participants were provided with information and all necessary material for the odor collection phase, which took place over five consecutive nights (see *Odor Sampling* for details). At the end of the odor collection phase, participants handed in their odor samples and were invited for a second lab appointment to take part in the actual experiment involving a standardized psychosocial stress task, the TSST^24, 38^.

Both partners within a couple were invited with an hour interval between appointments. The order of participation (woman – man vs. man – woman) was counterbalanced between couples. Experiments were scheduled during the luteal phase of the woman’s menstrual cycle (as confirmed by ovulation tests, taking place three days after a positive test at the earliest) but no later than three days prior to her next menstruation to avoid effects of premenstrual syndrome symptoms, and between 4.30 and 9.30 pm to control for circadian fluctuations of cortisol^38^. All participants were instructed to refrain from consuming alcohol, drugs, or psychoactive medication and to refrain from smoking for 24 h before the experiment. Moreover, they were instructed to shower with an unscented shower gel and to refrain from using any odorized products in the morning before testing, to minimize confounding odors during the experiment.

**Fig. 3:**
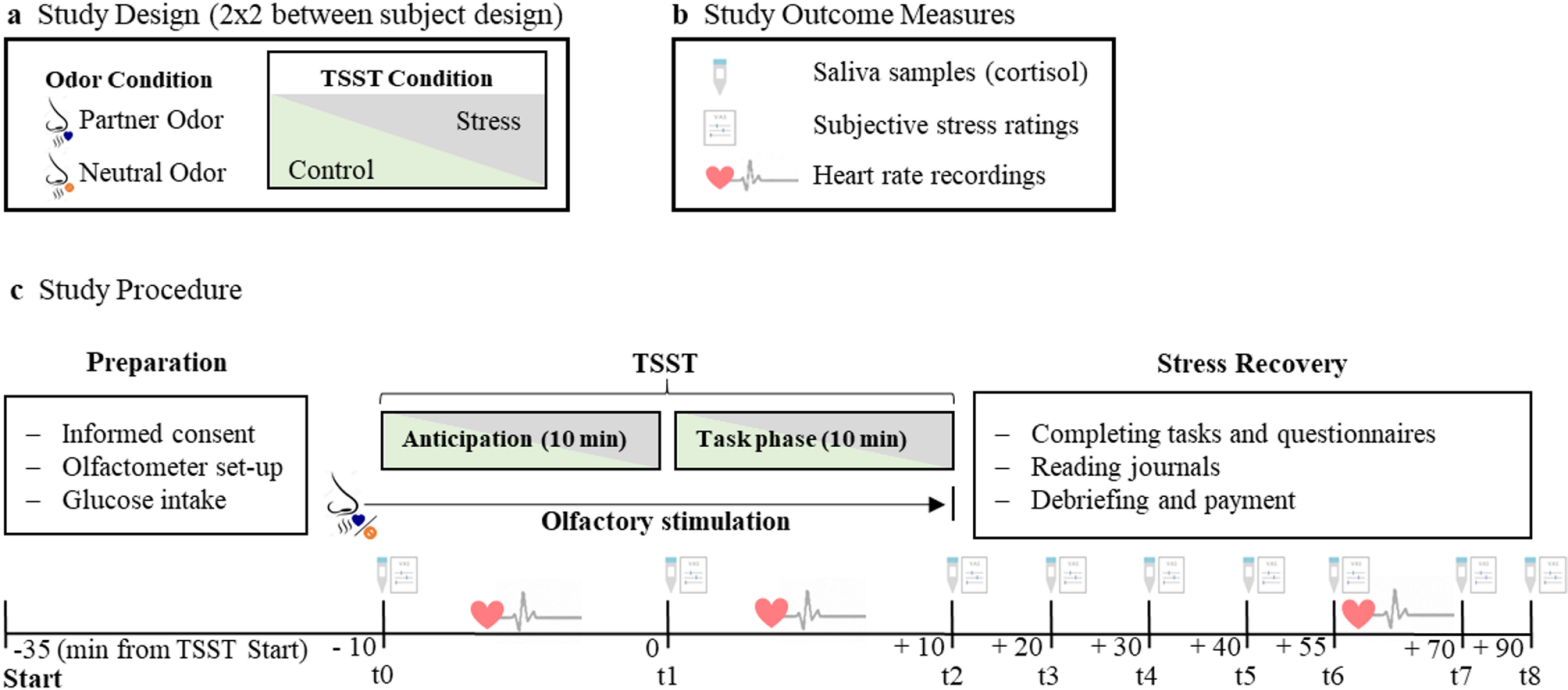
Overview of the study design (**a**), outcome measures (**b**) and experimental procedure (**c**).

For an overview of the procedure during the experimental session, see Fig. 3. During the experiment, participants were first prepared for and familiarized with all necessary equipment to conduct heart rate recordings and olfactory stimulation (see *Heart Rate Data collection*; *Olfactory Stimulation*). Next, they were instructed to consume 9 gram of glucose, to standardize blood sugar level^38^ and took either part in a stress inducing or a control version of the TSST (*see TSST*). Odor stimuli (partner odor or neutral non-human control odor) were presented over the whole TSST (including anticipation and task phase). After completion of the TSST participants were escorted to another room to accomplish an unrelated computer task, the details of which will be explored in future research. Throughout the experiment, we collected ratings of perceived stress, saliva samples (to assess cortisol response), and heart rate data (see *Stress Response Measures* and Fig. 3). Over the course of the experiment, participants filled in further questionnaires, a complete list of which can be found in the *Supplemental Materials F*. Upon completion, participants were debriefed by the experimenter and the TSST panel and received a monetary compensation of 110 € per person.

### Materials and instruments

#### Odor sampling

Participants collected their individual body odor at home by wearing odorless t-shirts (T-Shirt Slim Fit, 100% cotton, Trigema W. Grupp KG, Burladingen) with sewn-in cotton pads attached to both armpits on five consecutive nights, while sleeping alone in their bed. Female participants collected their body odor during the luteal phase of their menstrual cycle (as confirmed by ovulation tests, starting odor sampling three days after a positive test at the earliest) but no later than three days prior to their next menstruation, as men’s ratings concerning the attractiveness of women’s body odor have been shown to fluctuate systematically with the menstrual cycle^42^. Because cosmetic fragrances can influence social perceptions^43^, we aimed to minimize other confounding factors that might have altered the natural body odor: all participants were instructed to adhere to a set of rules over the entire time window of odor sampling (i.e., 5 days and nights), including to refrain from doing sports, consuming food known to influence one’s body odor (e.g. asparagus, garlic, spicy food, caffeine) or consuming drugs including cigarettes and alcohol, using any odorized products (e.g. perfume, deodorants, body lotion…), having sex while wearing the t-shirt, and from visiting places with strong smells (e.g. sauna, swimming pools). Moreover, participants were provided with unscented hygiene products (Balea Med ultra sensitive shampoo, shower gel, body lotion; dm-drogerie markt GmbH + Co. KG, Karlsruhe, Germany) and were instructed to wash their bedding, towels and all clothes in contact with the upper body with odorless washing powder prior to the odor sampling phase (Almawin & Klar, Ecosensitive unscented washing powder; naturPur Handels GmbH, Dudenhofen, Germany). During the day, participants were instructed to store the odor sampling t-shirts in airtight bags. At the end of the odor sampling phase, the participants returned their t-shirts to the laboratory. The two cotton pads in each shirt were removed, cut into four pieces each, and stored in individual airtight freezer bags at minus 80 °C^44^. During the whole procedure, the research assistant in charge wore a lab coat and gloves and used tweezers to lift up sampling pads. Furthermore, everyone involved in data collection and body odor sample processing was instructed to adhere to a large set of rules to avoid any external odors that might contaminate the body odor sample or the experimental situation. Odorless cotton pads, which underwent the same treatment, served as neutral control odors.

#### Odor presentation

At the day of the experiment, odor samples were put into airtight glass jars and thawed for approximately 60 min prior to the odor presentation. Median sample storage time in the freezer was 30 days (depending on the time period between odor sampling and experiment, both necessitating participants’ availability and a preceding positive ovulation test by the female participant). We used a computer-based sixteen-channel Lundström olfactometer (for technical details see^20^) for odor presentation, which allows for an accurate and reliable olfactory stimulation^45^, even below the threshold of conscious perception^46^. In the olfactometer, compressed air is filtered and odorized by passing through one of four odor reservoirs containing either the partner’s body odor (cotton pads cut into pieces) or neutral cotton pads without any body odor applied, while one-way valves prevent odor-contamination of the air tubes through air backflow. The odorized air then flows into a manifold, where it is combined with a continuous neutral airflow (0.5 l/m) to minimize distracting sensations due to a sudden flow onset. Finally, the odorized air is birhinally delivered into the participant’s left and right nostril via two nasal tubes at a calibrated total airflow rate of 3.00 l/m (+/- 0.05 l/m; resulting in 1.50 l per minute per nostril).

At the beginning of the experimental session, the participants were familiarized with the olfactometer. The manifold was attached to the participants’ breast via chest belts. Then nasal tubes were attached to the manifold and the participants were shown how to insert them into their nostrils. Finally, the airflow and odor presentation (in line with the experimental condition, i.e., partner odor vs. control odor) were demonstrated to participants for one minute. Subsequently, odor stimuli were continuously presented during the entire TSST (see Fig. 3), first with the manifold attached to the participants’ chest (anticipation phase) and next with the manifold positioned on a tripod stand, at an individually adjusted comfortable height (task phase). At the end of the experiment, participants provided feedback on how they experienced the olfactory stimulation procedure. We introduced post experimental ratings on the perceived intensity and pleasantness of the presented odor during the study. This addition did not affect other procedures; analyses found no significant differences in these subjective ratings between experimental conditions. Moreover, they rated their smelling abilities, their general perception of body odors and general associations with their partner’s body odor, including how sexy they perceived their partner’s body odor (for details and a full list of questionnaires and items see *Supplemental Materials F*).

#### TSST

The Trier Social Stress Test is a standard psychosocial stress induction task^24, 38^. In the TSST stress condition, participants were instructed to prepare a free speech for a mock job interview (10 min anticipation time). They were told that a panel of experts would evaluate their performance and that their interview would be videotaped. After the anticipation phase, the participants were guided to another room. There, they first had to give a free speech for five minutes and then solve difficult arithmetic problems for another five minutes. During the speech and the arithmetic task, a clearly visible camera recorded the participants. Participants had to perform the task in front of a panel consisting of a male and a female judge wearing white lab coats. Both judges were trained to maintain a neutral facial expression and to avoid any kind of verbal or non-verbal feedback. Additionally, the male judge gave instructions and interrupted participants during their free speech to increase stress if needed. Judges were rigorously trained to follow a strict protocol and keep interactions standardized. Previous studies have shown that this procedure validly and reliably evokes acute stress responses under experimental conditions^38, 47^, most prominently due to the perceived social-evaluative threat and uncontrollability of the situation^48^.

The procedure in the control condition^49^ was identical to that in the stress condition, except that social-evaluative threat was removed and the arithmetic problems were trivial instead of difficult. To achieve this, the participants were asked to prepare to talk aloud about their last holiday during the ten-minute anticipation phase. In addition, they were explicitly told that their performance would not be evaluated. The two parts of the task phase of the TSST were identical in length to those in the stress condition (five minutes of free speech and five minutes of arithmetic problems), but there were no judges or camera present.

#### Stress response measures

Perceived stress and the endocrine stress response were measured directly before the TSST-anticipation phase but after the first odor presentation (t_0_), after the TSST-anticipation phase (t_1_), after the TSST-task phase (t_2_), and at six time points during stress recovery (t_3 to 8_; for the time intervals (indicated as time since t_1_ in minutes) see Fig. 3). Heart rate data were sampled continuously over the course of the experiment. Due to participants moving around between different parts of the experiment, which could impact heart rate measurement, we only included heart rate data from the TSST-anticipation phase, the TSST-task phase and a resting phase at the end of the experiment between time points t_6_ and t_7_ . This interval was chosen to ensure sufficient recovery of baseline cortisol levels.

#### Perceived stress

Participants were asked to indicate how stressed they felt (“How stressed do you feel right now?”) on a visual analogue scale (VAS) from 0 (not at all) to 100 (very much). This item represents the primary stress measure of subjective stress^38, 50^. Additional VAS items assessed to what degree participants experienced physical discomfort, the desire to leave the situation, the desire for support, and feelings of control (reverse coded) in order to quantify stress related approach and avoidance tendencies^18^. Further information and supplemental findings on the composite stress responsiveness score, comprising all assessed VAS items can be found in the *Supplemental Materials G.*

#### Heart rate data collection

At the beginning of the experiment, participants were equipped with heart rate monitor watches and chest belt heart rate sensors (RS800CX, Polar Electro GmbH, Büttelborn, Germany). Over the course of the experiment, the experimenter instructed the participants to set individual time stamps on their heart rate monitor. After completion of data collection, all heart rate data were first visually inspected for technical failures and inconsistencies in time stamp assessment. Then, time intervals of interest were segmented (i.e., 10 min TSST-anticipation phase, 10 min TSST-task phase, and a 10 min non-stressful resting phase at the end of the experiment 50 min after the TSST, separated into five two-minute intervals for each phase) using Kubios HRV^51^ and artefact corrected data using a Kubios-integrated optimized correction algorithm. We used individual correction thresholds at the lowest possible level. No artifact correction was required for *n* = 85, the correction level ‘very low’ was selected for *n* = 21, the correction level ‘low’ for *n* = 19 and the correction level ‘medium’ for *n* = 16. An average of *M* = 7.19 (*SD* = 16.85) artifacts per participant were identified. Finally, we exported the calculated cardiovascular parameters for each interval of interest into .csv format for further analysis.

#### Cortisol

We collected saliva samples using SaliCaps (ultra-pure polypropylene tubes, IBL International GmbH) at nine time points throughout the experiment (see Fig. 1). Samples were stored at − 20 °C immediately after the experiment until cortisol levels could be determined. Salivary cortisol concentrations were determined by a commercially available chemiluminescence immunoassay (CLIA; IBL, Hamburg, Germany) at Dresden Lab Services, Dresden, Germany. For biochemical analyses, the samples were spun at 3000 rpm for 5 min to obtain clear saliva with low viscosity. Precision was good with inter and intra-assay coefficients of variation below 9%.

#### Questionnaires

Relevant variables, such as perceived daily stress, perceived chronic stress or trait anxiety were measured with validated questionnaires. Further information and a full list of applied questionnaires can be found in the *Supplemental Materials H.*

## Supplementary Information

Below is the link to the electronic supplementary material.

Supplementary Material 1

## Data Availability

All data, analysis code, and research materials are available at [https://osf.io/kc7qn/files/osfstorage? view_only=de978362db444f88a16e0d58893cb9f2](https:/osf.io/kc7qn/files/osfstorage? view_only=de978362db444f88a16e0d58893cb9f2).

## References

[1] Bratman, G. N. et al. Nature and human well-being: the olfactory pathway. *Sci. Adv.* **10**, eadn3028 (2024). 38748806 10.1126/sciadv.adn3028 PMC11809653

[2] Mahmut, M. K. & Croy, I. The role of body odors and olfactory ability in the initiation, maintenance and breakdown of romantic relationships–A review. *Physiol. Behav.* **207**, 179–184 (2019). 31077678 10.1016/j.physbeh.2019.05.003

[3] Lübke, K. T. & Pause, B. M. Always follow your nose: the functional significance of social chemosignals in human reproduction and survival. *Horm. Behav.* **68**, 134–144 (2015). 25637403 10.1016/j.yhbeh.2014.10.001

[4] Lundström, J. N. & Olsson, M. J. Functional neuronal processing of human body odors. *Vitam. Horm.* **83**, 1–23 (2010). 20831940 10.1016/S0083-6729(10)83001-8 PMC3593650

[5] Perl, O., Mishor, E., Ravia, A., Ravreby, I. & Sobel, N. Are humans constantly but subconsciously smelling themselves? *Philos. Trans. R Soc. B*. **375**, 20190372 (2020). 10.1098/rstb.2019.0372 PMC7209943 32306875

[6] Hofer, M. K., Chen, F. S. & Schaller, M. What your nose knows: Affective, cognitive, and behavioral responses to the scent of another person. *Curr. Dir. Psychol. Sci.* **29**, 617–623 (2020).

[7] McBurney, D. H., Shoup, M. L. & Streeter, S. A. Olfactory comfort: smelling a partner’s clothing during periods of separation. *J. Appl. Soc. Psychol.* **36**, 2325–2335 (2006).

[8] Havlicek, J. et al. He sees, she smells? Male and female reports of sensory reliance in mate choice and non-mate choice contexts. *Personal Individ Differ.* **45**, 565–570 (2008).

[9] Ditzen, B. et al. Effects of different kinds of couple interaction on cortisol and heart rate responses to stress in women. *Psychoneuroendocrinology* **32**, 565–574 (2007). 17499441 10.1016/j.psyneuen.2007.03.011

[10] Ditzen, B. & Heinrichs, M. Psychobiology of social support: the social dimension of stress buffering. *Restor. Neurol. Neurosci.* **32**, 149–162 (2014). 23603443 10.3233/RNN-139008

[11] Kirschbaum, C., Klauer, T., Filipp, S. H. & Hellhammer, D. H. Sex-specific effects of social support on cortisol and subjective responses to acute psychological stress. *Psychosom. Med.* **57**(1), 23–31 (1995). 7732155 10.1097/00006842-199501000-00004

[12] Uchino, B. N. & Way, B. M. Integrative pathways linking close family ties to health: A neurochemical perspective. *Am. Psychol.* **72**, 590–600 (2017). 28880105 10.1037/amp0000049

[13] Carpenter, E. M. & Kirkpatrick, L. A. Attachment style and presence of a romantic partner as moderators of Psychophysiological responses to a stressful laboratory situation. *Pers. Relatsh.* **3**, 351–367 (1996).

[14] Bourassa, K. J., Ruiz, J. M. & Sbarra, D. A. The impact of physical proximity and attachment working models on cardiovascular reactivity: comparing mental activation and romantic partner presence. *Psychophysiology* **56**, e13324 (2019). 30613999 10.1111/psyp.13324

[15] Kiyokawa, Y., Wakabayashi, Y., Takeuchi, Y. & Mori, Y. The neural pathway underlying social buffering of conditioned fear responses in male rats. *Eur. J. Neurosci.* **36**, 3429–3437 (2012). 22909130 10.1111/j.1460-9568.2012.08257.x

[16] Takahashi, Y. et al. Olfactory signals mediate social buffering of conditioned fear responses in male rats. *Behav. Brain Res.* **240**, 46–51 (2013). 23183219 10.1016/j.bbr.2012.11.017

[17] Hofer, M. K. & Chen, F. S. The scent of a good night’s sleep: olfactory cues of a romantic partner improve sleep efficiency. *Psychol. Sci.* **31**, 449–459 (2020). 32163721 10.1177/0956797620905615

[18] Hofer, M. K., Collins, H. K., Whillans, A. V. & Chen, F. S. Olfactory cues from romantic partners and strangers influence women’s responses to stress. *J. Pers. Soc. Psychol.* **114**, 1–9 (2018). 29293018 10.1037/pspa0000110

[19] Granqvist, P. et al. The scent of security: odor of romantic partner alters subjective discomfort and autonomic stress responses in an adult attachment-dependent manner. *Physiol. Behav.* **198**, 144–150 (2019). 30196084 10.1016/j.physbeh.2018.08.024

[20] Lundström, J. N., Gordon, A. R., Alden, E. C., Boesveldt, S. & Albrecht, J. Methods for Building an inexpensive computer-controlled olfactometer for temporally-precise experiments. *Int. J. Psychophysiol.* **78**, 179–189 (2010). 20688109 10.1016/j.ijpsycho.2010.07.007 PMC2967213

[21] Finke, J. B., Hahn, S., Schächinger, H. & Klucken, T. Increased pupil and heart-rate responses to sexual stimuli in men after physical exertion. *Psychophysiology* **60**, e14254 (2023). 36708087 10.1111/psyp.14254

[22] Goldey, K. L. & van Anders, S. M. Sexual thoughts: links to testosterone and cortisol in men. *Arch. Sex. Behav.* **41**, 1461–1470 (2012). 21993767 10.1007/s10508-011-9858-6

[23] Walla, P. et al. Evidence of conscious and subconscious olfactory information processing during word encoding: a magnetoencephalographic (MEG) study. *Cogn. Brain Res.* **14**, 309–316 (2002). 10.1016/s0926-6410(02)00121-0. 10.1016/s0926-6410(02)00121-0 12421654

[24] Kirschbaum, C., Pirke, K. M. & Hellhammer, D. H. The ‘Trier social stress Test’ – A tool for investigating Psychobiological stress responses in a laboratory setting. *Neuropsychobiology* **28**, 76–81 (1993). 8255414 10.1159/000119004

[25] Sorokowski, P. et al. Sex differences in human olfaction: a meta-analysis. *Front. Psychol.* **10**, 242 (2019). 30814965 10.3389/fpsyg.2019.00242 PMC6381007

[26] Bates, D., Mächler, M., Bolker, B. & Walker, S. Fitting linear Mixed-Effects models using ‘lme4’. *J. Stat. Softw.* **67**, 1–48 (2015).

[27] Kuznetsova, A., Brockhoff, P. B. & Christensen, R. H. B. LmerTest’ package: tests in linear mixed effects models. *J. Stat. Softw.* **82**, 1–26 (2017).

[28] Hollocks, M. J., Howlin, P., Papadopoulos, A. S., Khondoker, M. & Simonoff, E. Differences in HPA-axis and heart rate responsiveness to psychosocial stress in children with autism spectrum disorders with and without co-morbid anxiety. *Psychoneuroendocrinology* **46**, 32–45 (2014). 24882156 10.1016/j.psyneuen.2014.04.004

[29] Heinrichs, M., Baumgartner, T., Kirschbaum, C. & Ehlert, U. Social support and Oxytocin interact to suppress cortisol and subjective responses to psychosocial stress. *Biol. Psychiatry*. **54**, 1389–1398 (2003). 14675803 10.1016/s0006-3223(03)00465-7

[30] Bodenmann, G. et al. Effects of stress on the social support provided by men and women in intimate relationships. *Psychol. Sci.* **26**, 1584–1594 (2015). 26341561 10.1177/0956797615594616

[31] Campbell, J. & Ehlert, U. Acute psychosocial stress: Does the emotional stress response correspond with physiological responses?. *Psychoneuroendocrinology* **37**(8), 1111–1134 (2012). 22260938 10.1016/j.psyneuen.2011.12.010

[32] Prehn-Kristensen, A. et al. Induction of Empathy by the Smell of Anxiety. *PLOS ONE* **4**, e5987 (2009). 10.1371/journal.pone.0005987. 10.1371/journal.pone.0005987 PMC2695008 19551135

[33] Dutton, D. G. & Aron, A. P. Some evidence for heightened sexual attraction under conditions of high anxiety. *J. Pers. Soc. Psychol.* **30**, 510–517 (1974). 4455773 10.1037/h0037031

[34] March, D. S., Olson, M. A. & Fazio, R. H. The implicit misattribution model of evaluative conditioning. *Soc. Psychol. Bull.* **13**, 1–25 (2019).

[35] Zillmann, D. Excitation transfer theory in the international encyclopedia of communication (ed. Donsbach, W.) (2008) 10.1002/9781405186407.wbiece049

[36] Boyce, W. T. & Ellis, B. J. Biological sensitivity to context: I. An evolutionary–developmental theory of the origins and functions of stress reactivity. *Dev. Psychopathol.* **17**, 271–301 (2005). 16761546 10.1017/s0954579405050145

[37] Hahlweg, K. *Fragebogen Zur Partnerschaftsdiagnostik (FPD) [Marriage Diagnostic Questionnaire]* (Hogrefe, 1996).

[38] Labuschagne, I., Grace, C., Rendell, P., Terrett, G. & Heinrichs, M. An introductory guide to conducting the Trier social stress test. *Neurosci. Biobehav Rev.* **107**, 686–695 (2019). 31560923 10.1016/j.neubiorev.2019.09.032

[39] Pearlmutter, P. et al. Sweat and saliva cortisol response to stress and nutrition factors. *Sci. Rep.* **10**, 19050 (2020). 33149196 10.1038/s41598-020-75871-3 PMC7643128

[40] Hummel, T., Sekinger, B., Wolf, S. R., Pauli, E. & Kobal, G. Sniffin’sticks’: olfactory performance assessed by the combined testing of odor identification, odor discrimination and olfactory threshold. *Chem. Senses*. **22**, 39–52 (1997). 9056084 10.1093/chemse/22.1.39

[41] Hummel, T., Kobal, G., Gudziol, H. & Mackay-Sim, A. Normative data for the sniffin’ sticks including tests of odor identification, odor discrimination, and olfactory thresholds: an upgrade based on a group of more than 3,000 subjects. *Eur. Arch. Otorhinolaryngol.* **264**, 237–243 (2007). 17021776 10.1007/s00405-006-0173-0

[42] Gildersleeve, K. A., Haselton, M. G., Larson, C. M. & Pillsworth, E. G. Body odor attractiveness as a cue of impending ovulation in women: evidence from a study using hormone-confirmed ovulation. *Horm. Behav.* **61**, 157–166 (2012). 22137971 10.1016/j.yhbeh.2011.11.005

[43] Gaby, J. M., Gunaydin, G. & Zayas, V. The interactive role of odor associations in friendship preferences. *Sci. Rep.* **15**, 11228 (2025). 40175419 10.1038/s41598-025-94350-1 PMC11965504

[44] Lenochova, P., Roberts, S. C. & Havlicek, J. Methods of human body odor sampling: the effect of freezing. *Chem. Senses*. **34**, 127–138 (2009). 19005223 10.1093/chemse/bjn067

[45] Hayes, J. E., Jinks, A. L. & Stevenson, R. J. A comparison of Sniff bottle staircase and olfactometer-based threshold tests. *Behav. Res. Methods*. **45**, 178–182 (2013). 22836949 10.3758/s13428-012-0220-2

[46] Hummel, T., Olgun, S., Gerber, J., Huchel, U. & Frasnelli, J. Brain responses to odor mixtures with sub-threshold components. *Front. Psychol.* **4**, 786 (2013). 24167499 10.3389/fpsyg.2013.00786 PMC3807048

[47] Narvaez Linares, N. F., Charron, V., Ouimet, A. J., Labelle, P. R. & Plamondon, H. A systematic review of the Trier social stress test methodology: issues in promoting study comparison and replicable research. *Neurobiol. Stress*. **13**, 100235 (2020). 33344691 10.1016/j.ynstr.2020.100235 PMC7739033

[48] Dickerson, S. S. & Kemeny, M. E. Acute stressors and cortisol responses: A theoretical integration and synthesis of laboratory research. *Psychol. Bull.* **130**, 355–391 (2004). 15122924 10.1037/0033-2909.130.3.355

[49] Het, S., Rohleder, N., Schoofs, D., Kirschbaum, C. & Wolf, O. T. Neuroendocrine and psychometric evaluation of a placebo version of the ‘Trier social stress test’. *Psychoneuroendocrinology* **34**, 1075–1086 (2009). 19307062 10.1016/j.psyneuen.2009.02.008

[50] von Dawans, B., Fischbacher, U., Kirschbaum, C., Fehr, E. & Heinrichs, M. The social dimension of stress reactivity: acute stress increases prosocial behavior in humans. *Psychol. Sci.* **23**, 651–660 (2012). 22593119 10.1177/0956797611431576

[51] Tarvainen, M. P., Niskanen, J. P., Lipponen, J. A., Ranta-aho, P. O. & Karjalainen, P. A. Kubios HRV – Heart rate variability analysis software. *Comput. Methods Programs Biomed.* **113**, 210–220 (2014). 24054542 10.1016/j.cmpb.2013.07.024

