# Supplementary material for: Smelling the romantic partner’s natural body odor increases psychological and autonomic but not cortisol stress responses
Source: Sci Rep. 2026 Jan 14;16:1760. doi: 10.1038/s41598-025-27639-w (PMC12804818; doi:10.1038/s41598-025-27639-w)
Supplement: Supplementary file 1 — Supplementary Material 1 [file 41598_2025_27639_MOESM1_ESM.docx]

**Supplemental Materials**

**Table of Contents**

A: Analyses with Covariates2

B: Analyses with Sex as Moderator6

C: Correlations of Stress Indicators13

D: Analysis with Sexual Attractiveness – Full Sample 15

E: Additional Variables Related to Sexual Attractiveness18

F: Study Materials32

G: Composite Stress Measure Analyses49

H: Participant Flow52

References53

**A: Analyses with Covariates**

We conducted analyses for the central dependent variables: perceived stress (see Table A1), cortisol (See Table A2), and heart rates (see Table A3) also with the following control variables included: trait anxiety as measured with the state-trait anxiety inventory ^1^, perceived daily stress measured with the PSS-10 ^2^, and chronic stress measured with the TICS ^3^, none of these variables significantly influences the outcome variables and the analyses yield the same pattern of results as those reported in the main text.

**Table A1**

Mixed linear effects model predicting perceived stress.

| *Predictors* | | *b* | | *SE(b)* | *CI* | *t* | | *df* | *p* |
| --- | --- | --- | --- | --- | --- | --- | --- | --- | --- |
| Intercept | | 11.54 | | 5.02 | 1.65 – 21.43 | 2.30 | | 218.53 | .022 |
| TSST condition¹ | | -7.97 | | 3.80 | -15.44 – -0.50 | -2.10 | | 463.07 | .037 |
| Odor² | | -6.64 | | 3.81 | -14.13 – 0.85 | -1.74 | | 473.47 | .082 |
| Time segment 1³ | | -1.70 | | 1.21 | -4.08 – 0.67 | -1.41 | | 1412.22 | .160 |
| Time segment 2⁴ | | -1.16 | | 0.38 | -1.90 – -0.42 | -3.08 | | 1412.75 | .002 |
| Sex⁵ | | 5.13 | | 2.05 | 1.08 – 9.19 | 2.50 | | 171.18 | .013 |
| Trait Anxiety⁶ | | 0.31 | | 0.17 | -0.03 – 0.64 | 1.82 | | 170.92 | .071 |
| Perceived Daily Stress⁷ | | 0.16 | | 0.27 | -0.37 – 0.70 | 0.61 | | 171.08 | .541 |
| Chronic Stress⁸ | | -0.01 | | 0.21 | -0.41 – 0.40 | -0.03 | | 171.02 | .977 |
| TSST condition¹ × Odor² | | 15.97 | | 5.38 | 5.39 – 26.55 | 2.97 | | 471.82 | .003 |
| Time segment 1³ × TSST condition¹ | | 7.89 | | 1.72 | 4.51 – 11.28 | 4.58 | | 1412.34 | <.001 |
| Time segment 1³ × Odor² | | 2.08 | | 1.74 | -1.34 – 5.50 | 1.19 | | 1412.20 | .232 |
| Time segment 2⁴ × TSST condition¹ | | -2.45 | | 0.54 | -3.51 – -1.40 | -4.57 | | 1413.06 | <.001 |
| Time segment 2⁴ × Odor² | | 0.13 | | 0.54 | -0.93 – 1.19 | 0.24 | | 1412.61 | .813 |
| Time segment 1³ × TSST condition¹ × Odor² | | -0.77 | | 2.46 | -5.59 – 4.05 | -0.31 | | 1412.27 | .754 |
| Time segment 2⁴ × TSST condition¹ × Odor² | | -1.59 | | 0.76 | -3.09 – -0.10 | -2.09 | | 1412.81 | .037 |
| **Random Effects** | | | | | | | | | |
| σ^2^ | 190.80 | | τ_00_ _ID_ | | 162.76 | | ICC | | .46 |
| Observations (*N*_ID_ = 179) | | | | 1599 | | | | | |
| Marginal R^2^ / Conditional R^2^ | | | | .173 / .554 | | | | | |

*Note.* ¹ control = 0, stress = 1, ² control = 0, partner = 1, ³ breakpoint at t_2_: t_0_ = 0, t_1_ = 1, t_2_ to t_8_ = 2, ⁴ breakpoint at t_2_: t_0_ to t_2_ = 0, t_3_ = 1, t_4_ = 2, t_5_ = 3, t_6_ = 4, t_7_ = 5, t_8_ = 6, ⁵ male = 0, female = 1, ⁶ STAI-trait, ⁷ PSS, ⁸ TICS.

**Table A2**

Mixed linear effects model predicting Cortisol levels.

| *Predictors* | | *b* | *SE(b)* | *CI* | *t* | *df* | *p* |
| --- | --- | --- | --- | --- | --- | --- | --- |
| Intercept | | 2.55 | 0.73 | 1.10 – 3.99 | 3.47 | 186.54 | .001 |
| TSST condition¹ | | -0.34 | 0.49 | -1.31 – 0.63 | -0.69 | 259.16 | .493 |
| Odor² | | -0.07 | 0.49 | -1.04 – 0.90 | -0.14 | 262.19 | .891 |
| Time segment 1³ | | -0.26 | 0.06 | -0.38 – -0.14 | -4.12 | 1422.98 | <.001 |
| Time segment 2⁴ | | -0.07 | 0.06 | -0.20 – 0.05 | -1.17 | 1422.98 | .244 |
| Sex⁵ | | -0.76 | 0.31 | -1.38 – -0.15 | -2.45 | 170.98 | .015 |
| Trait Anxiety⁶ | | -0.02 | 0.03 | -0.07 – 0.03 | -0.77 | 170.96 | .443 |
| Perceived Daily Stress⁷ | | 0.04 | 0.04 | -0.04 – 0.12 | 0.93 | 170.96 | .355 |
| Chronic Stress⁸ | | 0.01 | 0.03 | -0.06 – 0.07 | 0.17 | 170.96 | .867 |
| TSST condition¹ × Odor² | | 0.06 | 0.70 | -1.31 – 1.43 | 0.09 | 261.61 | .932 |
| Time segment 1³ × TSST condition¹ | | 1.42 | 0.09 | 1.24 – 1.60 | 15.88 | 1422.98 | <.001 |
| Time segment 1³ × Odor² | | 0.05 | 0.09 | -0.13 – 0.23 | 0.53 | 1422.98 | .599 |
| Time segment 2⁴ × TSST condition¹ | | -0.96 | 0.09 | -1.14 – -0.79 | -10.74 | 1423.08 | <.001 |
| Time segment 2⁴ × Odor² | | -0.02 | 0.09 | -0.20 – 0.15 | -0.27 | 1422.98 | .787 |
| Time segment 1³ × TSST condition¹ × Odor² | | -0.10 | 0.13 | -0.35 – 0.15 | -0.81 | 1422.98 | .419 |
| Time segment 2⁴ × TSST condition¹ × Odor² | | -0.05 | 0.13 | -0.30 – 0.20 | -0.42 | 1423.03 | .678 |
| **Random Effects** | | | | | | | |
| σ^2^ | 2.25 | | τ_00_ _ID_ | 4.00 | | ICC | .64 |
| Observations (*N*_ID_ = 179) | | 1610 | | | | | |
| Marginal R^2^ / Conditional R^2^ | | .318 / .755 | | | | | |

*Note.* ¹ control = 0, stress = 1, ² control = 0, partner = 1, ³ breakpoint at t_4_: t_0_ = 0, t_1_ = 1, t_2_ = 2, t_3_ = 3, t_4_ to t_8_ = 4, ⁴ breakpoint at t_4_: t_0_ to t_4_ = 0, t_5_ = 1, t_6_ = 2, t_7_ = 3, t_8_ = 4, ⁵ male = 0, female = 1, ⁶ STAI-trait, ⁷ PSS, ⁸ TICS.

**Table A3**

Mixed linear effects model predicting heart rates.

| *Predictors* | | *b* | *SE(b)* | *CI* | *t* | *df* | *p* |
| --- | --- | --- | --- | --- | --- | --- | --- |
| Intercept | | 59.70 | 4.82 | 50.18 – 69.22 | 12.40 | 140.24 | <.001 |
| TSST condition¹ | | 3.37 | 2.91 | -2.38 – 9.11 | 1.16 | 151.01 | .249 |
| Odor condition² | | 1.20 | 2.95 | -4.62 – 7.03 | 0.41 | 151.42 | .683 |
| Anticipation phase³ | | 7.76 | 0.75 | 6.30 – 9.23 | 10.39 | 2036.00 | <.001 |
| TSST phase⁴ | | 17.40 | 0.75 | 15.94 – 18.87 | 23.28 | 2036.00 | <.001 |
| Sex⁵ | | 6.55 | 2.03 | 2.54 – 10.56 | 3.23 | 138.00 | .002 |
| Trait Anxiety⁶ | | 0.13 | 0.17 | -0.20 – 0.47 | 0.78 | 138.00 | .435 |
| Perceived Daily Stress⁷ | | -0.17 | 0.27 | -0.70 – 0.36 | -0.63 | 138.00 | .528 |
| Chronic Stress⁸ | | 0.03 | 0.20 | -0.36 – 0.43 | 0.17 | 138.00 | .863 |
| TSST condition¹ × Odor² | | -0.63 | 4.15 | -8.84 – 7.58 | -0.15 | 151.31 | .880 |
| TSST condition¹ × Anticipation phase³ | | 5.97 | 1.06 | 3.89 – 8.04 | 5.64 | 2036.00 | <.001 |
| TSST condition¹ × TSST phase⁴ | | 10.62 | 1.06 | 8.55 – 12.70 | 10.05 | 2036.00 | <.001 |
| Odor condition² × Anticipation phase³ | | 3.01 | 1.09 | 0.87 – 5.14 | 2.76 | 2036.00 | .006 |
| Odor² × TSST phase⁴ | | 4.02 | 1.09 | 1.89 – 6.16 | 3.70 | 2036.00 | <.001 |
| TSST condition¹ × Odor² × Anticipation phase³ | | 0.76 | 1.53 | -2.23 – 3.76 | 0.50 | 2036.00 | .617 |
| TSST condition¹ × Odor² × TSST phase⁴ | | 5.60 | 1.53 | 2.61 – 8.60 | 3.67 | 2036.00 | <.001 |
| **Random Effects** | | | | | | | |
| σ^2^ | 53.11 | τ_00_ _ID_ | | 141.11 | | ICC | 0.73 |
| Observations (*N*_ID_ = 146) | | 2190 | | | | | |
| Marginal R^2^ / Conditional R^2^ | | .455 / .851 | | | | | |

*Note.* ¹ control = 0, stress = 1, ² control = 0, partner = 1, ³ dummy coded, anticipation phase = 1, resting phase = 0, TSST phase = 0, ⁴ dummy coded, TSST phase = 1, anticipation phase = 0, resting phase = 0, ⁵ male = 0, female = 1, ⁶ STAI-trait, ⁷ PSS, ⁸ TICS..

**B: Analyses with Sex as Moderator**

To explore whether sex might moderate the effect of olfactory presence on stress responses, we performed additional analyses for our primary outcome measures: perceived stress (see Table E1), cortisol (see Table B2), and heart rates (see Table B3). The analyses included the same predictors as those reported in the main text, but additionally all interaction terms (two-way, three-way, and four-way) between sex and the other predictors. We found no significant interaction with sex in the analyses.

Consistent with prior research, women reported higher perceived stress ^4^, had higher heart rates ^5^, and their cortisol levels were lower ^6^ – see the main paper for main-effect models. However, sex did not significantly moderate the effects of partner odor. Larger samples and additional studies will be needed to investigate sex differences.

**Table B1**

Mixed linear effects model predicting perceived stress.

| *Predictors* | *b* | *SE(b)* | *CI* | *t* | *df* | *p* |
| --- | --- | --- | --- | --- | --- | --- |
| Intercept | 21.85 | 3.77 | 14.44 – 29.26 | 5.79 | 460.12 | <.001 |
| TSST condition¹ | -4.53 | 5.23 | -14.81 – 5.74 | -0.87 | 460.12 | .386 |
| Odor ² | 1.58 | 5.39 | -9.02 – 12.18 | 0.29 | 460.11 | .769 |
| Time segment 1³ | -1.67 | 1.70 | -5.01 – 1.67 | -0.98 | 1404.20 | .326 |
| Time segment 2⁴ | -0.48 | 0.53 | -1.52 – 0.56 | -0.90 | 1404.72 | .367 |
| Sex⁵ | 11.24 | 5.33 | 0.75 – 21.72 | 2.11 | 460.13 | .036 |
| TSST condition¹ × Odor² | 9.42 | 7.77 | -5.85 – 24.69 | 1.21 | 460.12 | .226 |
| Time segment 1³ × TSST condition¹ | 8.49 | 2.36 | 3.86 – 13.12 | 3.60 | 1404.20 | <.001 |
| Time segment 1³ × Odor² | -0.80 | 2.44 | -5.58 – 3.98 | -0.33 | 1404.18 | .742 |
| TSST condition¹ × Sex⁵ | -6.12 | 7.61 | -21.07 – 8.84 | -0.80 | 460.20 | .422 |
| Odor² × Sex⁵ | -14.87 | 7.68 | -29.95 – 0.22 | -1.94 | 460.12 | .053 |
| Time segment 1³ × Sex⁵ | -0.06 | 2.41 | -4.78 – 4.67 | -0.02 | 1404.22 | .981 |
| Time segment 2⁴ × TSST condition¹ | -2.47 | 0.73 | -3.91 – -1.03 | -3.37 | 1404.70 | .001 |
| Time segment 2⁴ × Odor² | -0.60 | 0.75 | -2.08 – 0.88 | -0.80 | 1404.43 | .424 |
| Time segment 2⁴ × Sex⁵ | -1.37 | 0.75 | -2.83 – 0.10 | -1.82 | 1404.72 | .068 |
| Time segment 1³ × TSST condition¹ × Odor² | -0.26 | 3.51 | -7.14 – 6.63 | -0.07 | 1404.19 | .942 |
| TSST condition¹ × Odor² × Sex⁵ | 12.91 | 10.90 | -8.50 – 34.32 | 1.18 | 460.41 | .237 |
| Time segment 1³ × TSST condition¹ × Sex⁵ | -1.31 | 3.44 | -8.07 – 5.45 | -0.38 | 1404.36 | .704 |
| Time segment 1³ × Odor² × Sex⁵ | 5.89 | 3.47 | -0.91 – 12.69 | 1.70 | 1404.20 | .089 |

**Table B1 (continued)**

| *Predictors* | *b* | | *SE(b)* | *CI* | *t* | *df* | *p* |
| --- | --- | --- | --- | --- | --- | --- | --- |
| Time segment 2⁴ × TSST condition¹ × Odor² | -1.36 | | 1.09 | -3.49 – 0.78 | -1.25 | 1404.62 | .213 |
| Time segment 2⁴ × TSST condition¹ × Sex⁵ | -0.17 | | 1.07 | -2.28 – 1.94 | -0.16 | 1405.10 | .875 |
| Time segment 2⁴ × Odor² × Sex⁵ | 1.48 | | 1.08 | -0.63 – 3.59 | 1.37 | 1404.59 | .170 |
| Time segment 1³ × TSST condition¹ × Odor² × Sex⁵ | -1.62 | | 4.92 | -11.28 – 8.04 | -0.33 | 1404.28 | .742 |
| Time segment 2⁴ × TSST condition¹ × Odor² × Sex⁵ | -0.23 | | 1.53 | -3.23 – 2.78 | -0.15 | 1404.84 | .883 |
| **Random Effects** | | | | | | | |
| σ^2^ | 188.87 | | | | | | |
| τ_00_ _ID_ | 172.91 | | | | | | |
| ICC | .48 | | | | | | |
| Observations (*N*_ID_ = 179) | | 1599 | | | | | |
| Marginal R^2^ / Conditional R^2^ | | 0.156 / 0.560 | | | | | |

*Note.* ¹ control = 0, stress = 1, ² control = 0, partner = 1, ³ breakpoint at t_2_: t_0_ = 0, t_1_ = 1, t_2_ to t_8_ = 2, ⁴ breakpoint at t_2_: t_0_ to t_2_ = 0, t_3_ = 1, t_4_ = 2, t_5_ = 3, t_6_ = 4, t_7_ = 5, t_8_ = 6, ⁵ male = 0, female = 1.

**Table B2**

Mixed linear effects model predicting cortisol.

| *Predictors* | *b* | *SE(b)* | *CI* | *t* | *df* | *p* |
| --- | --- | --- | --- | --- | --- | --- |
| Intercept | 2.05 | 0.48 | 1.11 – 3.00 | 4.29 | 264.42 | <.001 |
| TSST condition¹ | 0.15 | 0.66 | -1.15 – 1.46 | 0.23 | 264.42 | .816 |
| Odor ² | 0.46 | 0.68 | -0.89 – 1.81 | 0.67 | 264.42 | .505 |
| Time segment 1³ | -0.19 | 0.09 | -0.36 – -0.02 | -2.14 | 1414.98 | .033 |
| Time segment 2⁴ | -0.10 | 0.09 | -0.28 – 0.07 | -1.14 | 1414.98 | .253 |
| Sex⁵ | 0.27 | 0.68 | -1.06 – 1.60 | 0.40 | 264.42 | .691 |
| TSST condition¹ × Odor² | -0.49 | 0.99 | -2.43 – 1.46 | -0.49 | 264.42 | .622 |
| Time segment 1³ × TSST condition¹ | 1.47 | 0.12 | 1.23 – 1.71 | 11.94 | 1414.98 | <.001 |
| Time segment 1³ × Odor² | -0.06 | 0.13 | -0.31 – 0.19 | -0.48 | 1414.98 | .628 |
| TSST condition¹ × Sex⁵ | -1.12 | 0.97 | -3.02 – 0.78 | -1.16 | 264.42 | .247 |
| Odor² × Sex⁵ | -1.08 | 0.97 | -3.00 – 0.83 | -1.11 | 264.42 | .267 |
| Time segment 1³ × Sex⁵ | -0.14 | 0.13 | -0.38 – 0.11 | -1.11 | 1414.98 | .268 |
| Time segment 2⁴ × TSST condition¹ | -1.10 | 0.12 | -1.34 – -0.86 | -8.97 | 1414.98 | <.001 |
| Time segment 2⁴ × Odor² | -0.02 | 0.13 | -0.27 – 0.23 | -0.14 | 1414.98 | .885 |
| Time segment 2⁴ × Sex⁵ | 0.06 | 0.13 | -0.19 – 0.30 | 0.45 | 1414.98 | .655 |
| Time segment 1³ × TSST condition¹ × Odor² | 0.06 | 0.18 | -0.30 – 0.42 | 0.34 | 1414.98 | .732 |
| TSST condition¹ × Odor² × Sex⁵ | 1.26 | 1.38 | -1.46 – 3.99 | 0.91 | 264.42 | .362 |
| Time segment 1³ × TSST condition¹ × Sex⁵ | -0.12 | 0.18 | -0.47 – 0.23 | -0.68 | 1414.99 | .494 |
| Time segment 1³ × Odor² × Sex⁵ | 0.22 | 0.18 | -0.13 – 0.57 | 1.22 | 1414.98 | .223 |
| Time segment 2⁴ × TSST condition¹ × Odor² | -0.06 | 0.18 | -0.42 – 0.30 | -0.33 | 1414.98 | .744 |

**Table B2 (continued)**

| *Predictors* | | *b* | *SE(b)* | *CI* | *t* | *df* | *p* |
| --- | --- | --- | --- | --- | --- | --- | --- |
| Time segment 2⁴ × TSST condition¹ × Sex⁵ | 0.32 | | 0.18 | -0.03 – 0.68 | 1.80 | 1415.11 | .072 |
| Time segment 2⁴ × Odor² × Sex⁵ | -0.01 | | 0.18 | -0.36 – 0.34 | -0.06 | 1414.98 | .951 |
| Time segment 1³ × TSST condition¹ × Odor² × Sex⁵ | -0.25 | | 0.26 | -0.75 – 0.26 | -0.96 | 1414.99 | .337 |
| Time segment 2⁴ × TSST condition¹ × Odor² × Sex⁵ | -0.09 | | 0.26 | -0.59 – 0.41 | -0.35 | 1415.05 | .727 |
| **Random Effects** | | | | | | | |
| σ^2^ | | 2.23 | | | | | |
| τ_00_ _ID_ | | 3.98 | | | | | |
| ICC | | 0.64 | | | | | |
| Observations (*N*_ID_ = 179) | | 1610 | | | | | |
| Marginal R^2^ / Conditional R^2^ | | .323 / .756 | | | | | |

*Note.* ¹ control = 0, stress = 1, ² control = 0, partner = 1, ³ breakpoint at t_4_: t_0_ = 0, t_1_ = 1, t_2_ = 2, t_3_ = 3, t_4_ to t_8_ = 4, ⁴ breakpoint at t_4_: t_0_ to t_4_ = 0, t_5_ = 1, t_6_ = 2, t_7_ = 3, t_8_ = 4, ⁵ male = 0, female = 1.

**Table B3**

Mixed linear effects model predicting heart rates.

| *Predictors* | *b* | *SE(b)* | *CI* | *t* | *df* | *p* |
| --- | --- | --- | --- | --- | --- | --- |
| Intercept | 63.73 | 3.00 | 57.80 – 69.65 | 21.25 | 151.45 | <.001 |
| TSST condition¹ | 3.13 | 3.99 | -4.76 – 11.02 | 0.78 | 151.45 | .434 |
| Odor² | 1.07 | 4.24 | -7.31 – 9.45 | 0.25 | 151.45 | .801 |
| Anticipation phase³ | 6.56 | 1.11 | 4.38 – 8.73 | 5.92 | 2028.00 | <.001 |
| TSST phase⁴ | 14.69 | 1.11 | 12.52 – 16.86 | 13.26 | 2028.00 | <.001 |
| Sex⁵ | 4.92 | 4.03 | -3.05 – 12.89 | 1.22 | 151.45 | .225 |
| TSST condition¹ × Odor² | 1.09 | 5.93 | -10.62 – 12.80 | 0.18 | 151.45 | .855 |
| TSST condition¹ × Anticipation phase³ | 7.43 | 1.48 | 4.53 – 10.32 | 5.04 | 2028.00 | <.001 |
| TSST condition¹ × TSST phase⁴ | 11.18 | 1.48 | 8.29 – 14.08 | 7.58 | 2028.00 | <.001 |
| Odor condition² × Anticipation phase³ | 3.10 | 1.57 | 0.03 – 6.17 | 1.98 | 2028.00 | .048 |
| Odor condition² × TSST phase⁴ | 5.48 | 1.57 | 2.41 – 8.55 | 3.50 | 2028.00 | <.001 |
| TSST condition¹ × Sex⁵ | -0.04 | 5.73 | -11.35 – 11.27 | -0.01 | 151.45 | .994 |
| Odor condition² × Sex⁵ | 0.20 | 5.85 | -11.37 – 11.76 | 0.03 | 151.45 | .973 |
| Anticipation phase³ × Sex⁵ | 2.19 | 1.49 | -0.73 – 5.11 | 1.47 | 2028.00 | .142 |
| TSST phase⁴ × Sex⁵ | 4.91 | 1.49 | 1.98 – 7.83 | 3.29 | 2028.00 | .001 |
| TSST condition¹ × Odor² × Anticipation phase³ | -2.41 | 2.19 | -6.70 – 1.88 | -1.10 | 2028.00 | .271 |
| TSST condition¹ × Odor² × TSST phase⁴ | 3.43 | 2.19 | -0.86 – 7.72 | 1.57 | 2028.00 | .117 |
| TSST condition¹ × Odor² × Sex⁵ | -2.57 | 8.26 | -18.90 – 13.75 | -0.31 | 151.45 | .756 |
| TSST condition¹ × Anticipation phase³ × Sex⁵ | -2.78 | 2.12 | -6.93 – 1.37 | -1.32 | 2028.00 | .188 |

**Table B3 (continued)**

| *Predictors* | *b* | *SE(b)* | *CI* | *t* | *df* | *p* |
| --- | --- | --- | --- | --- | --- | --- |
| TSST condition¹ × TSST phase⁴ × Sex⁵ | 0.21 | 2.12 | -3.94 – 4.36 | 0.10 | 2028.00 | .922 |
| Odor² × Anticipation phase³ × Sex⁵ | 0.04 | 2.16 | -4.20 – 4.28 | 0.02 | 2028.00 | .986 |
| Odor² × TSST phase⁴ × Sex⁵ | -2.39 | 2.16 | -6.63 – 1.85 | -1.11 | 2028.00 | .268 |
| TSST condition¹ × Odor² × Anticipation phase³ × Sex⁵ | 5.40 | 3.05 | -0.58 – 11.39 | 1.77 | 2028.00 | .077 |
| TSST condition¹ × Odor² × TSST phase⁴ × Sex⁵ | 2.20 | 3.05 | -3.78 – 8.19 | 0.72 | 2028.00 | .471 |
| **Random Effects** | | | | | | |
| σ^2^ | 52.17 | | | | | |
| τ_00_ _ID_ | 154.46 | | | | | |
| ICC | 0.73 | | | | | |
| Observations (*N*_ID_ = 146) | 2160 | | | | | |
| Marginal R^2^ / Conditional R^2^ | .456 / .854 | | | | | |

*Note.* ¹ control = 0, stress = 1, ² control = 0, partner = 1, ³ dummy coded, anticipation phase = 1, resting phase = 0, TSST phase = 0, ⁴ dummy coded, TSST phase = 1, anticipation phase = 0, resting phase = 0, ⁵ male = 0, female = 1.

**C: Correlations of Stress Indicators**

Perceived stress during the anticipation phase (t_1_) was positively correlated with cortisol levels approximately 20 minutes later (t_3_), *r* = .25, *t*(176) = 3.34, *p* < .001. Similarly, perceived stress during the TSST-task phase (t_2_) was positively correlated with cortisol levels 20 minutes later (t_4_), *r* = .21, *t*(177) = 2.86, *p* = .005. Average heart rates in the anticipation phase and the TSST-task phase were positively correlated with perceived stress measured at the same time intervals: *r* = .33, *t*(143) = 4.26, *p* < .001 (anticipation) and *r* = .27, *t*(144) = 3.37, *p* < .001 (TSST-task). We also observed positive correlations between average heart rates and the cortisol response about 20 minutes later: for heart rates measured during the anticipation phase with cortisol at t_3_, *r* = .35, *t*(144) = 4.51, *p* < .001, and for heart rates measured during the TSST-task phase and cortisol measured at t_4_, *r* = .38, *t*(144) = 4.94, *p* < .001. For a full summary of the correlations, see Table C1.

**Table C1**

*Means, standard deviations, and correlations with confidence intervals*

| Variable | *M* | *SD* | 1 | 2 | 3 | 4 | 5 |
| --- | --- | --- | --- | --- | --- | --- | --- |
|  |  |  |  |  |  |  |  |
| 1. Perceived Stress t_1_ | 31.11 | 20.12 |  |  |  |  |  |
|  |  |  |  |  |  |  |  |
| 2. Perceived Stress t_2_ | 35.32 | 23.81 | .67** |  |  |  |  |
|  |  |  | [.58, .75] |  |  |  |  |
|  |  |  |  |  |  |  |  |
| 3. Cortisol t_3_ | 3.61 | 3.77 | .25** | .16* |  |  |  |
|  |  |  | [.11, .38] | [.02, .30] |  |  |  |
|  |  |  |  |  |  |  |  |
| 4. Cortisol t_4_ | 3.87 | 4.37 | .23** | .21** | .93** |  |  |
|  |  |  | [.08, .36] | [.07, .35] | [.90, .95] |  |  |
|  |  |  |  |  |  |  |  |
| 5. Mean Heart Rate t_1_ | 80.71 | 14.93 | .34** | .13 | .35** | .30** |  |
|  |  |  | [.18, .47] | [-.04, .28] | [.20, .49] | [.14, .44] |  |
|  |  |  |  |  |  |  |  |
| 6. Mean Heart Rate t_2_ | 94.39 | 19.15 | .31** | .27** | .38** | .38** | .83** |
|  |  |  | [.15, .45] | [.11, .41] | [.23, .51] | [.23, .51] | [.77, .88] |
|  |  |  |  |  |  |  |  |

*Note.* *M* and *SD* are used to represent mean and standard deviation, respectively. Values in square brackets indicate the 95% confidence interval for each correlation. * indicates *p* < .05. ** indicates *p* < .01.

**D: Analysis with Sexual Attractiveness – Full Sample**

To explore the role of sexual attraction induced heart rate increases, we analyzed the conditional effect of odor on heart rates moderated by the individual rating each participant gave about their partner odor’s sexual attractiveness. The model is summarized in Table D1. We found significant four-way interactions between the TSST condition, odor, the phase variables, and sexual attractiveness, indicating that the conditional odor effects differed between TSST conditions, particularly during the TSST-phase.

The odor dependent relation between sexual attractiveness and heart rates was stronger in the TSST-condition during the TSST-task phase. In other words, especially when participants were under acute stress (i.e., in the TSST condition and the TSST-task phase), did we observe that odor sexual attractiveness positively predicted heart rates. It is possible that acute stress might increase sexual attraction-induced heart rate changes, since previous studies have found that physical exertion increased sexual arousal for men ^7^, but for women, decreased sexual arousal was reported under psychosocial stress ^8^. Examining a potential higher-order interaction with sex goes beyond the present paper – the resulting five-way interactions would most likely not be properly interpretable. Additional studies particularly designed to examine stress effects on arousal would be needed to address whether they are sex-dependent.

**Table D1**

Mixed linear effects model predicting heart rates.

| Predictors | *b* | *SE(b)* | *CI* | *t* | *df* | *p* |
| --- | --- | --- | --- | --- | --- | --- |
| Intercept | 62.58 | 2.30 | 58.04 – 67.13 | 27.20 | 148.07 | <.001 |
| TSST condition¹ | 3.53 | 2.87 | -2.13 – 9.20 | 1.23 | 151.03 | .220 |
| Odor² | 1.57 | 2.92 | -4.19 – 7.34 | 0.54 | 151.09 | .590 |
| Anticipation phase³ | 6.95 | 0.78 | 5.43 – 8.48 | 8.93 | 2028.00 | <.001 |
| TSST phase⁴ | 16.05 | 0.78 | 14.52 – 17.57 | 20.62 | 2028.00 | <.001 |
| Sexual attractiveness⁵ | 1.33 | 2.07 | -2.77 – 5.43 | 0.64 | 151.00 | .523 |
| Sex⁶ | 6.25 | 1.97 | 2.35 – 10.14 | 3.17 | 137.00 | .002 |
| TSST condition¹ × Odor² | -0.40 | 4.09 | -8.48 – 7.69 | -0.10 | 150.98 | .923 |
| TSST condition¹ × Anticipation phase³ | 6.70 | 1.08 | 4.58 – 8.83 | 6.18 | 2028.00 | <.001 |
| TSST condition¹ × TSST phase⁴ | 11.66 | 1.08 | 9.53 – 13.78 | 10.75 | 2028.00 | <.001 |
| Odor condition² × Anticipation phase³ | 3.70 | 1.11 | 1.53 – 5.87 | 3.35 | 2028.00 | .001 |
| Odor condition² × TSST phase⁴ | 5.38 | 1.11 | 3.21 – 7.54 | 4.86 | 2028.00 | <.001 |
| TSST condition¹ × Sexual attractiveness⁵ | -2.06 | 2.74 | -7.47 – 3.35 | -0.75 | 151.08 | .453 |
| Odor condition² × Sexual attractiveness⁵ | -0.36 | 3.17 | -6.62 – 5.90 | -0.11 | 151.10 | .910 |
| Sexual attractiveness⁵ × Anticipation phase³ | 2.61 | 0.78 | 1.07 – 4.15 | 3.33 | 2028.00 | .001 |
| Sexual attractiveness ⁵ × TSST phase⁴ | 4.36 | 0.78 | 2.83 – 5.90 | 5.57 | 2028.00 | <.001 |
| TSST condition¹ × Odor² × Anticipation phase³ | 0.46 | 1.54 | -2.57 – 3.49 | 0.30 | 2028.00 | .765 |
| TSST condition¹ × Odor² × TSST phase⁴ | 5.06 | 1.54 | 2.03 – 8.09 | 3.27 | 2028.00 | .001 |
| TSST condition¹ × Odor² × Sexual attractiveness⁵ | 3.95 | 4.17 | -4.28 – 12.18 | 0.95 | 151.10 | .344 |
| TSST condition¹ × Anticipation phase³ × Sexual attractiveness⁵ | -2.94 | 1.04 | -4.97 – -0.90 | -2.83 | 2028.00 | .005 |

**Table D1 (continued)**

| Predictors | *b* | *SE(b)* | *CI* | *t* | *df* | *p* |
| --- | --- | --- | --- | --- | --- | --- |
| TSST ¹ × TSST phase⁴ × Sexual attractiveness⁵ | -5.79 | 1.04 | -7.83 – -3.76 | -5.58 | 2028.00 | <.001 |
| Odor² × Anticipation phase³ × Sexual attractiveness⁵ | -1.15 | 1.20 | -3.51 – 1.20 | -0.96 | 2028.00 | .338 |
| Odor² × TSST phase⁴ × Sexual attractiveness⁵ | -4.30 | 1.20 | -6.66 – -1.95 | -3.58 | 2028.00 | <.001 |
| TSST condition¹ × Odor² × Anticipation phase³ × Sexual attractiveness⁵ | 3.44 | 1.58 | 0.34 – 6.53 | 2.18 | 2028.00 | .030 |
| TSST condition¹ × Odor² × TSST phase⁴ × Sexual attractiveness⁵ | 8.68 | 1.58 | 5.58 – 11.78 | 5.50 | 2028.00 | <.001 |
| Random Effects | | | | | | |
| σ^2^ | 51.92 | | | | | |
| τ_00_ _ID_ | 134.18 | | | | | |
| ICC | .72 | | | | | |
| Observations (*N*_ID_ = 146) | 2190 | | | | | |
| Marginal *R*^2^ / Conditional *R*^2^ | .481 / .855 | | | | | |

*Note.* ¹ control = 0, stress = 1, ² control = 0, partner = 1, ³ dummy coded, anticipation phase = 1, resting phase = 0, TSST phase = 0, ⁴ dummy coded, TSST phase = 1, anticipation phase = 0, resting phase = 0, ⁵ z-standardized, ⁶ male = 0, female = 1.

**E: Additional Variables Related to Sexual Attractiveness**

In addition to the rated general sexual attractiveness of one’s partner’s odor, we used three different indicators related to sexual attraction as moderators to investigate the interplay between odor stimuli and sexual attraction induced changes in heart rate.

*First*, participants’ frequency of sexual intercourse – assuming that those participants who often engage in sex with their partner also associate their partners odor more with sexual attraction and arousal. For a summary of the model for the full sample see Table E1. The overall pattern is similar to that observed with the rated sexual attractiveness as the moderator – when odor was presented, the heart rates of those participants in the TSST-stress condition were positively related to their frequency of sexual intercourse. This relation was especially pronounced in the TSST-task phase. This interpretation is further supported by an analysis focusing only on those participants in the TSST-stress condition (see Table E2 for a summary and Figure E1 for a visualization).

*Second*, participants’ rating of general loveliness of their partner’s odor (item “I love my partner’s odor.”). For a summary of the full model see Table E3. For an analysis focusing on the TSST-stress condition see Table E4 and Figure E2. These analyses revealed a similar pattern. Again, the loveliness rating positively predicted heart rates if the odor was presented, especially when acute stress was induced.

*Third*, participants rated how pleasant the presented odor stimulus was. This variable was added later in the post experimental questionnaire and data are only available for 60 participant (31 in the TSST-stress condition). The findings using this variable are summarized in Table E5 for the full sample and in Table H6 for those in the TSST-stress condition. For a visualization see Figure E3.

Across the three measures, we find a conditional odor effect that seems to be dependent on attraction. It should be noted that these analyses rely on vastly different operationalizations of the odor attraction link. While the sexual attractiveness rating reported in the main text and the loveliness rating discussed in these supplemental analyses refer to general ratings of the partners’ odor (not the presented stimuli), frequency of sexual intercourse does not refer to odor at all (but it is reasonable to assume that those participants who have more sex also associate their partner’s odor more strongly with sexual arousal and attraction), and the rating of the presented stimulus is decoupled from conscious partner associations, because participants were not aware of the origin of the stimuli. This converging evidence speaks for a role of sexual attraction in bodily reactions to partner odor.

**Table E1**

Mixed linear effects model predicting heart rates.

| Predictors | *b* | *SE*(*b*) | *CI* | *t* | *df* | *p* |
| --- | --- | --- | --- | --- | --- | --- |
| Intercept | 62.89 | 2.31 | 58.32 – 67.46 | 27.20 | 147.01 | <.001 |
| TSST condition¹ | 3.51 | 2.87 | -2.16 – 9.17 | 1.22 | 150.21 | .223 |
| Odor condition² | 0.85 | 3.02 | -5.11 – 6.82 | 0.28 | 150.30 | .779 |
| Anticipation phase³ | 7.78 | 0.75 | 6.32 – 9.24 | 10.43 | 2028.00 | <.001 |
| TSST phase⁴ | 17.45 | 0.75 | 15.99 – 18.91 | 23.40 | 2028.00 | <.001 |
| Sex frequency⁵ | -0.34 | 1.81 | -3.92 – 3.23 | -0.19 | 150.33 | .850 |
| Sex⁶ | 6.36 | 2.04 | 2.32 – 10.39 | 3.12 | 137.00 | .002 |
| TSST condition¹ × Odor condition² | -0.48 | 4.20 | -8.78 – 7.81 | -0.12 | 150.16 | .908 |
| TSST condition¹ × Anticipation phase³ | 6.01 | 1.05 | 3.94 – 8.08 | 5.70 | 2028.00 | <.001 |
| TSST condition¹ × TSST phase⁴ | 10.47 | 1.05 | 8.40 – 12.53 | 9.93 | 2028.00 | <.001 |
| Odor condition² × Anticipation phase³ | 3.32 | 1.11 | 1.14 – 5.51 | 2.98 | 2028.00 | .003 |
| Odor condition² × TSST phase⁴ | 5.06 | 1.11 | 2.88 – 7.25 | 4.55 | 2028.00 | <.001 |
| TSST condition¹ × Sex frequency⁵ | 1.83 | 2.68 | -3.47 – 7.13 | 0.68 | 150.33 | .497 |
| Odor condition² × Sex frequency⁵ | 1.97 | 3.23 | -4.41 – 8.34 | 0.61 | 150.31 | .543 |
| Sex frequency⁵ × Anticipation phase³ | 0.13 | 0.67 | -1.18 – 1.44 | 0.19 | 2028.00 | .848 |
| Sex frequency⁵ × TSST phase⁴ | 0.38 | 0.67 | -0.94 – 1.69 | 0.56 | 2028.00 | .574 |
| TSST condition¹ × Odor condition² × Anticipation phase³ | 0.40 | 1.54 | -2.62 – 3.42 | 0.26 | 2028.00 | .796 |
| TSST condition¹ × Odor condition² × TSST phase⁴ | 4.69 | 1.54 | 1.67 – 7.72 | 3.05 | 2028.00 | .002 |

**Table E1 (continued)**

| Predictors | *b* | *SE*(*b*) | *CI* | *t* | *df* | *p* |
| --- | --- | --- | --- | --- | --- | --- |
| TSST condition¹ × Odor condition² × Sex frequency⁵ | -3.97 | 4.32 | -12.51 – 4.58 | -0.92 | 150.31 | .360 |
| TSST condition¹ × Anticipation phase³ × Sex frequency⁵ | 0.41 | 0.99 | -1.53 – 2.36 | 0.42 | 2028.00 | .677 |
| TSST condition¹ × TSST phase⁴ × Sex frequency⁵ | -1.40 | 0.99 | -3.34 – 0.54 | -1.41 | 2028.00 | .157 |
| Odor condition² × Anticipation phase³ × Sex frequency⁵ | -1.37 | 1.19 | -3.70 – 0.97 | -1.15 | 2028.00 | .251 |
| Odor condition² × TSST phase⁴ × Sex frequency⁵ | -4.42 | 1.19 | -6.75 – -2.08 | -3.71 | 2028.00 | <.001 |
| TSST condition¹ × Odor condition² × Anticipation phase³ × Sex frequency⁵ | 2.96 | 1.60 | -0.17 – 6.09 | 1.86 | 2028.00 | .064 |
| TSST condition¹ × Odor condition² × TSST phase⁴ × Sex frequency⁵ | 9.07 | 1.60 | 5.94 – 12.20 | 5.68 | 2028.00 | <.001 |
| **Random Effects** | | | | | | |
| σ^2^ | 52.16 | | | | | |
| τ_00_ _ID_ | 142.59 | | | | | |
| ICC | .73 | | | | | |
| Observations (*N*_ID_ = 146) | 2190 | | | | | |
| Marginal R^2^ / Conditional R^2^ | .457 / .855 | | | | | |

*Note.* ¹ control = 0, stress = 1, ² control = 0, partner = 1, ³ dummy coded, anticipation phase = 1, resting phase = 0, TSST phase = 0, ⁴ dummy coded, TSST phase = 1, anticipation phase = 0, resting phase = 0, ⁵ z-standardized, ⁶ male = 0, female = 1.

**Table E2**

Mixed linear effects model predicting heart rates in the TSST-stress subgroup.

| *Predictors* | *b* | *SE(b)* | *CI* | *t* | *df* | *p* |
| --- | --- | --- | --- | --- | --- | --- |
| Intercept | 66.60 | 2.48 | 61.66 – 71.53 | 26.88 | 74.71 | <.001 |
| Odor condition¹ | 0.59 | 3.07 | -5.54 – 6.71 | 0.19 | 76.77 | .849 |
| Anticipation phase² | 13.76 | 0.85 | 12.10 – 15.42 | 16.24 | 1028.00 | <.001 |
| TSST phase³ | 27.98 | 0.85 | 26.31 – 29.64 | 33.01 | 1028.00 | <.001 |
| Sex frequency⁴ | 1.49 | 2.09 | -2.68 – 5.66 | 0.71 | 76.98 | .480 |
| Sex⁵ | 5.68 | 3.05 | -0.40 – 11.77 | 1.86 | 69.00 | .067 |
| Odor condition¹ × Anticipation phase² | 3.63 | 1.22 | 1.25 – 6.01 | 2.99 | 1028.00 | .003 |
| Odor condition¹ × TSST phase³ | 9.49 | 1.22 | 7.10 – 11.87 | 7.81 | 1028.00 | <.001 |
| Odor condition¹ × Sex frequency⁴ | -1.91 | 3.08 | -8.03 – 4.22 | -0.62 | 76.79 | .537 |
| Sex frequency⁴ × Anticipation phase² | 0.54 | 0.84 | -1.10 – 2.19 | 0.65 | 1028.00 | .517 |
| Sex frequency⁴ × TSST phase³ | -1.03 | 0.84 | -2.67 – 0.61 | -1.23 | 1028.00 | .220 |
| Odor condition¹ × Anticipation phase² × Sex frequency⁴ | 1.60 | 1.22 | -0.79 – 3.99 | 1.31 | 1028.00 | .189 |
| Odor condition¹ × TSST phase³ × Sex frequency⁴ | 4.67 | 1.22 | 2.28 – 7.06 | 3.84 | 1028.00 | <.001 |
| **Random Effects** | | | | | | |
| σ^2^ | 68.07 | | | | | |
| τ_00_ _ID_ | 156.58 | | | | | |
| ICC | .70 | | | | | |
| Observations (*N*_ID_ = 74) | 1110 | | | | | |
| Marginal R^2^ / Conditional R^2^ | .476 / .841 | | | | | |

*Note.* ¹ control = 0, partner = 1, ² dummy coded, anticipation phase = 1, resting phase = 0, TSST phase = 0, ³ dummy coded, TSST phase = 1, anticipation phase = 0, resting phase = 0, ⁴ z-standardized, ⁵ male = 0, female = 1.

**Table E3**

Mixed linear effects model predicting heart rates.

| Predictors | *b* | *SE*(*b*) | *CI* | *t* | *df* | *p* |
| --- | --- | --- | --- | --- | --- | --- |
| Intercept | 62.61 | 2.30 | 58.07 – 67.16 | 27.21 | 147.70 | <.001 |
| TSST condition¹ | 3.79 | 2.86 | -1.87 – 9.44 | 1.32 | 150.79 | .188 |
| Odor condition² | 1.39 | 2.93 | -4.40 – 7.19 | 0.47 | 150.86 | .635 |
| Anticipation phase³ | 7.43 | 0.77 | 5.93 – 8.93 | 9.70 | 2028.00 | <.001 |
| TSST phase⁴ | 16.94 | 0.77 | 15.44 – 18.44 | 22.11 | 2028.00 | <.001 |
| Loveliness rating⁵ | 1.21 | 2.21 | -3.16 – 5.57 | 0.55 | 150.79 | .586 |
| Sex⁶ | 6.46 | 2.00 | 2.51 – 10.41 | 3.23 | 137.00 | .002 |
| TSST condition¹ × Odor condition² | -0.69 | 4.12 | -8.83 – 7.46 | -0.17 | 150.75 | .868 |
| TSST condition¹ × Anticipation phase³ | 6.05 | 1.07 | 3.94 – 8.16 | 5.63 | 2028.00 | <.001 |
| TSST condition¹ × TSST phase⁴ | 10.81 | 1.07 | 8.70 – 12.92 | 10.07 | 2028.00 | <.001 |
| Odor condition² × Anticipation phase³ | 3.16 | 1.10 | 0.99 – 5.32 | 2.86 | 2028.00 | .004 |
| Odor condition² × TSST phase⁴ | 4.37 | 1.10 | 2.21 – 6.54 | 3.96 | 2028.00 | <.001 |
| TSST condition¹ × Loveliness rating⁵ | 0.35 | 3.05 | -5.67 – 6.37 | 0.11 | 150.87 | .910 |
| Odor condition² × Loveliness rating⁵ | -0.17 | 3.26 | -6.61 – 6.27 | -0.05 | 150.81 | .959 |
| Loveliness rating⁵ × Anticipation phase³ | 1.54 | 0.83 | -0.08 – 3.17 | 1.86 | 2028.00 | .063 |
| Loveliness rating⁵ × TSST phase⁴ | 2.14 | 0.83 | 0.52 – 3.77 | 2.58 | 2028.00 | .010 |
| TSST condition¹ × Odor condition² × Anticipation phase³ | 1.05 | 1.54 | -1.98 – 4.08 | 0.68 | 2028.00 | .498 |
| TSST condition¹ × Odor condition² × TSST phase⁴ | 5.81 | 1.54 | 2.78 – 8.84 | 3.76 | 2028.00 | <.001 |

**Table E3 (continued)**

| Predictors | *b* | *SE*(*b*) | *CI* | *t* | *df* | *p* |
| --- | --- | --- | --- | --- | --- | --- |
| TSST condition¹ × Odor condition² × Loveliness rating⁵ | 0.68 | 4.23 | -7.68 – 9.04 | 0.16 | 150.87 | .873 |
| TSST condition¹ × Anticipation phase³ × Loveliness rating⁵ | -3.42 | 1.15 | -5.67 – -1.18 | -2.99 | 2028.00 | .003 |
| TSST condition¹ × TSST phase⁴ × Loveliness rating⁵ | -4.23 | 1.15 | -6.48 – -1.98 | -3.69 | 2028.00 | <.001 |
| Odor condition² × Anticipation phase³ × Loveliness rating⁵ | 0.08 | 1.22 | -2.32 – 2.48 | 0.06 | 2028.00 | .949 |
| Odor condition² × TSST phase⁴ × Loveliness rating⁵ | -1.11 | 1.22 | -3.51 – 1.29 | -0.91 | 2028.00 | .365 |
| TSST condition¹ × Odor condition² × Anticipation phase³ × Loveliness rating⁵ | 2.74 | 1.59 | -0.38 – 5.86 | 1.72 | 2028.00 | .085 |
| TSST condition¹ × Odor condition² × TSST phase⁴ × Loveliness rating⁵ | 4.63 | 1.59 | 1.50 – 7.75 | 2.91 | 2028.00 | .004 |
| **Random Effects** | | | | | | |
| σ^2^ | 52.70 | | | | | |
| τ_00_ _ID_ | 138.48 | | | | | |
| ICC | 0.72 | | | | | |
| Observations (*N*_ID_ = 146) | 2190 | | | | | |
| Marginal R^2^ / Conditional R^2^ | 0.467 / 0.853 | | | | | |

*Note.* ¹ control = 0, stress = 1, ² control = 0, partner = 1, ³ dummy coded, anticipation phase = 1, resting phase = 0, TSST phase = 0, ⁴ dummy coded, TSST phase = 1, anticipation phase = 0, resting phase = 0, ⁵ z-standardized, ⁶ male = 0, female = 1.

**Table E4**

Mixed linear effects model predicting heart rates in the TSST-stress subgroup.

| *Predictors* | *b* | *SE(b)* | *CI* | *t* | *df* | *p* |
| --- | --- | --- | --- | --- | --- | --- |
| Intercept | 66.25 | 2.43 | 61.40 – 71.10 | 27.21 | 74.97 | <.001 |
| Odor condition¹ | 0.66 | 3.04 | -5.39 – 6.72 | 0.22 | 77.04 | .828 |
| Anticipation phase² | 13.79 | 0.85 | 12.12 – 15.46 | 16.20 | 1028.00 | <.001 |
| TSST phase³ | 28.09 | 0.85 | 26.42 – 29.76 | 33.00 | 1028.00 | <.001 |
| Loveliness rating⁴ | 1.68 | 2.39 | -3.08 – 6.45 | 0.70 | 77.19 | .484 |
| Sex⁵ | 6.21 | 2.97 | 0.29 – 12.14 | 2.09 | 69.00 | .040 |
| Odor condition¹ × Anticipation phase² | 3.74 | 1.22 | 1.35 – 6.14 | 3.07 | 1028.00 | .002 |
| Odor condition¹ × TSST phase³ | 9.61 | 1.22 | 7.22 – 12.01 | 7.88 | 1028.00 | <.001 |
| Odor condition¹ × Loveliness rating⁴ | 0.52 | 3.08 | -5.61 – 6.66 | 0.17 | 77.19 | .865 |
| Loveliness rating⁴ × Anticipation phase² | -2.02 | 0.97 | -3.92 – -0.12 | -2.08 | 1028.00 | .037 |
| Loveliness rating⁴ × TSST phase³ | -2.24 | 0.97 | -4.14 – -0.34 | -2.31 | 1028.00 | .021 |
| Odor condition¹ × Anticipation phase² × Loveliness rating⁴ | 3.02 | 1.25 | 0.58 – 5.47 | 2.43 | 1028.00 | .015 |
| Odor condition¹ × TSST phase³ × Loveliness rating⁴ | 3.77 | 1.25 | 1.32 – 6.22 | 3.02 | 1028.00 | .003 |
| **Random Effects** | | | | | | |
| σ^2^ | 68.75 | | | | | |
| τ_00_ _ID_ | 152.58 | | | | | |
| ICC | .69 | | | | | |
| Observations (*N*_ID_ = 74) | 1110 | | | | | |
| Marginal R^2^ / Conditional R^2^ | .483 / .839 | | | | | |

*Note.* ¹ control = 0, partner = 1, ² dummy coded, anticipation phase = 1, resting phase = 0, TSST phase = 0, ³ dummy coded, TSST phase = 1, anticipation phase = 0, resting phase = 0, ⁴ z-standardized, ⁵ male = 0, female = 1.

**Table E5**

Mixed linear effects model predicting heart rates.

| Predictors | *b* | *SE*(*b*) | *CI* | *t* | *df* | *p* |
| --- | --- | --- | --- | --- | --- | --- |
| Intercept | 61.83 | 4.57 | 52.68 – 70.98 | 13.54 | 55.18 | <.001 |
| TSST condition¹ | 4.77 | 5.46 | -6.17 – 15.71 | 0.87 | 56.29 | .386 |
| Odor condition² | 1.58 | 5.21 | -8.86 – 12.02 | 0.30 | 56.55 | .763 |
| Anticipation phase³ | 5.58 | 1.35 | 2.93 – 8.24 | 4.13 | 838.00 | <.001 |
| TSST phase⁴ | 17.29 | 1.35 | 14.63 – 19.94 | 12.78 | 838.00 | <.001 |
| Pleasantness rating⁵ | 2.29 | 5.83 | -9.38 – 13.96 | 0.39 | 56.57 | .696 |
| Sex⁶ | 8.62 | 3.74 | 1.11 – 16.13 | 2.30 | 52.00 | .025 |
| TSST condition¹ × Odor condition² | -0.03 | 7.49 | -15.02 – 14.96 | -0.00 | 56.36 | .997 |
| TSST condition¹ × Anticipation phase³ | 8.86 | 1.87 | 5.20 – 12.52 | 4.75 | 838.00 | <.001 |
| TSST condition¹ × TSST phase⁴ | 14.64 | 1.87 | 10.97 – 18.30 | 7.84 | 838.00 | <.001 |
| Odor condition² × Anticipation phase³ | 4.29 | 1.83 | 0.69 – 7.88 | 2.34 | 838.00 | .020 |
| Odor condition² × TSST phase⁴ | 3.70 | 1.83 | 0.10 – 7.29 | 2.02 | 838.00 | .044 |
| TSST condition¹ × Pleasantness rating⁵ | -1.29 | 6.67 | -14.64 – 12.06 | -0.19 | 56.63 | .847 |
| Odor condition² × Pleasantness rating⁵ | -3.60 | 6.87 | -17.37 – 10.17 | -0.52 | 56.51 | .602 |
| Pleasantness rating⁵ × Anticipation phase³ | -1.18 | 2.05 | -5.20 – 2.84 | -0.58 | 838.00 | .565 |
| Pleasantness rating⁵ × TSST phase⁴ | -2.89 | 2.05 | -6.91 – 1.13 | -1.41 | 838.00 | .159 |
| TSST condition¹ × Odor condition² × Anticipation phase³ | 1.24 | 2.58 | -3.82 – 6.29 | 0.48 | 838.00 | .632 |
| TSST condition¹ × Odor condition² × TSST phase⁴ | 3.43 | 2.58 | -1.63 – 8.49 | 1.33 | 838.00 | .184 |

**Table E5 (continued)**

| Predictors | *b* | *SE*(*b*) | *CI* | *t* | *df* | *p* |
| --- | --- | --- | --- | --- | --- | --- |
| TSST condition¹ × Odor condition² × Pleasantness rating⁵ | 7.78 | 8.18 | -8.60 – 24.17 | 0.95 | 56.62 | .345 |
| TSST condition¹ × Anticipation phase³ × Pleasantness rating⁵ | -1.81 | 2.36 | -6.45 – 2.82 | -0.77 | 838.00 | .442 |
| TSST condition¹ × TSST phase⁴ × Pleasantness rating⁵ | -0.55 | 2.36 | -5.18 – 4.08 | -0.23 | 838.00 | .816 |
| Odor condition² × Anticipation phase³ × Pleasantness rating⁵ | 2.62 | 2.40 | -2.09 – 7.34 | 1.09 | 838.00 | .275 |
| Odor condition² × TSST phase⁴ × Pleasantness rating⁵ | 5.32 | 2.40 | 0.60 – 10.04 | 2.21 | 838.00 | .027 |
| TSST condition¹ × Odor condition² × Anticipation phase³ × Pleasantness rating⁵ | 2.09 | 2.89 | -3.59 – 7.77 | 0.72 | 838.00 | .470 |
| TSST condition¹ × Odor condition² × TSST phase⁴ × Pleasantness rating⁵ | 2.01 | 2.89 | -3.67 – 7.69 | 0.69 | 838.00 | .488 |
| **Random Effects** | | | | | | |
| σ^2^ | 61.02 | | | | | |
| τ_00_ _ID_ | 182.38 | | | | | |
| ICC | .75 | | | | | |
| Observations (*N*_ID_ = 61) | 915 | | | | | |
| Marginal R^2^ / Conditional R^2^ | 0.462 / 0.865 | | | | | |

*Note.* ¹ control = 0, stress = 1, ² control = 0, partner = 1, ³ dummy coded, anticipation phase = 1, resting phase = 0, TSST phase = 0, ⁴ dummy coded, TSST phase = 1, anticipation phase = 0, resting phase = 0, ⁵ z-standardized, ⁶ male = 0, female = 1.

**Table E6**

Mixed linear effects model predicting heart rates in the TSST-stress subgroup.

| *Predictors* | *b* | *SE(b)* | *CI* | *t* | *df* | *p* |
| --- | --- | --- | --- | --- | --- | --- |
| Intercept | 66.56 | 3.68 | 59.02 – 74.10 | 18.08 | 28.49 | <.001 |
| Odor condition¹ | 1.24 | 4.90 | -8.79 – 11.27 | 0.25 | 28.73 | .802 |
| Anticipation phase² | 14.69 | 1.35 | 12.04 – 17.34 | 10.90 | 426.00 | <.001 |
| TSST phase³ | 32.21 | 1.35 | 29.56 – 34.86 | 23.89 | 426.00 | <.001 |
| Pleasantness rating⁴ | 1.10 | 3.49 | -6.04 – 8.24 | 0.31 | 28.85 | .755 |
| Sex⁵ | 8.47 | 4.91 | -1.62 – 18.57 | 1.73 | 26.00 | .096 |
| Odor condition¹ × Anticipation phase² | 5.13 | 1.87 | 1.44 – 8.81 | 2.74 | 426.00 | .006 |
| Odor condition¹ × TSST phase³ | 6.51 | 1.87 | 2.83 – 10.19 | 3.47 | 426.00 | .001 |
| Odor condition¹ × Pleasantness rating⁴ | 4.74 | 4.83 | -5.15 – 14.62 | 0.98 | 28.82 | .335 |
| Pleasantness rating⁴ × Anticipation phase² | -3.37 | 1.36 | -6.04 – -0.69 | -2.47 | 426.00 | .014 |
| Pleasantness rating⁴ × TSST phase³ | -3.86 | 1.36 | -6.54 – -1.19 | -2.84 | 426.00 | .005 |
| Odor condition¹ × Anticipation phase² × Pleasantness rating⁴ | 5.30 | 1.88 | 1.61 – 8.99 | 2.83 | 426.00 | .005 |
| Odor condition¹ × TSST phase³ × Pleasantness rating⁴ | 8.24 | 1.88 | 4.55 – 11.93 | 4.39 | 426.00 | <.001 |
| **Random Effects** | | | | | | |
| σ^2^ | 65.48 | | | | | |
| τ_00_ _ID_ | 152.02 | | | | | |
| ICC | 0.70 | | | | | |
| Observations (*N*_ID_ = 31) | 465 | | | | | |
| Marginal R^2^ / Conditional R^2^ | 0.556 / 0.866 | | | | | |

*Note.* ¹ control = 0, partner = 1, ² dummy coded, anticipation phase = 1, resting phase = 0, TSST phase = 0, ³ dummy coded, TSST phase = 1, anticipation phase = 0, resting phase = 0, ⁴ z-standardized, ⁵ male = 0, female = 1.

**Figure E1**

Heart rates in the TSST-stress condition by phase and frequency of sexual intercourse

*Note.* Dots represent raw data points. Solid lines show the prediction of the mixed model with 95% confidence intervals as shaded areas.

**Figure E2**

Heart rates in the TSST-stress condition by phase and general partner odor loveliness.

*Note.* Dots represent raw data points. Solid lines show the prediction of the mixed model with 95% confidence intervals as shaded areas.

**Figure E3**

Heart rates in the TSST-stress condition by phase and presented odor pleasantness.

*Note.* Dots represent raw data points. Solid lines show the prediction of the mixed model with 95% confidence intervals as shaded areas.

**F: Study Materials**

Below is a list of items originally created for this study that were presented to participants during the pre-screening and the main session (translated into English). All items are listed in the order they were assessed. References for validated questionnaires used in the present study are provided and indicated in italics.

**Prescreening**

| **Item** | **Response options** |
| --- | --- |
| Age | open response (numeric, in years) |
| Sex | - male - female |
| Native language | - German - other (open response for language) |
| Height in cm | open response (numeric) |
| Weight in kg | open response (numeric) |
| Marital status | - single - married - divorced - widowed |
| Are you currently in a committed relationship? | - yes - no |
| For how long have you been in this relationship? | open responses for years and month (numeric) |
| Do you use hormonal contraception?  (e.g. pill, contraceptive patch, contraceptive stick, vaginal ring, hormonal coil, three-monthly injection) | - yes - no |
| If yes: Which hormonal contraceptive method do you use? | - Pill - Vaginal ring - Contraceptive patch - Contraceptive sticks - Hormone coil - Three-monthly injection |
| Do you smoke? | - yes - no - now and then |
| If yes: How often do you smoke? | - several times a week - once a week - less than once a week - once a month - less than once a month |
| Do you have a pet? | - yes - no |
| If yes: What kind of pets do you have? | open response (text) |
| Years of education | open response (numeric) |
| Highest level of education | - No degree or certificate - Primary school/basic high school - Secondary education with a high school diploma - Vocational diploma - Abitur (university entrance qualification) - University degree |
| Occupational status | - Vocational student - College/University student - Employed - Self-employed - Retired - Looking for work |
| If employed: Occupation | open response |
| If student: Field(s) of study | open response |
| Do you have a part-time job? | - yes - no |
| Do you work in shifts? | - yes - no |
| If yes: When do you work in shift work? | - only during the day - only at night - alternating day and night |
| Please enter a valid telephone number where we can reach you. | open response |
| Please enter a valid e-mail address where we can reach you. | open response |
| *If participants passed all inclusion checks, they were asked the Marriage Diagnostic Questionnaire (Hahlweg, 1996), commonly used instrument to assess relationship quality, that can also be used for unmarried couples.* | Hahlweg, K. (1996). *Fragebogen zur Partnerschaftsdiagnostik* (FPD). Hogrefe Verlag für Psychologie*.* |
| Next, we will contact you by telephone.  Please let us know 1-2 possible dates or a time of day that is convenient for you when we can easily reach you at the number given. | open response |

**Questions asked during the experiment**

The following set of questions were asked at nine time points (t_0_ to t_8_, see Figure 1 in the manuscript) to assess relevant states. The very last item refers directly to the stress experienced by the participants and is the primary indicator of perceived stress.

| **Item** | **Response options** |
| --- | --- |
| How strong is your desire to have a familiar person by your side right now? | Visual analogue scale from 0 “no desire” to 100 “very strong desire” |
| To what extent are you feeling physically uncomfortable right now? | Visual analogue scale from 0 “not uncomfortable” to 100 “very uncomfortable” |
| How strong is your desire to leave this situation? | Visual analogue scale from 0 “not at all” to 100 “very strong” |
| To what extent do you feel in control of the situation? | Visual analogue scale from 0 “not at all” to 100 “very much” |
| To what extent do you feel stressed? | Visual analogue scale from 0 “not at all” to 100 “very much” |
| *For exploratory purposes at some of the measurements during the experiment state anxiety* ^1^ *was also assessed after perceived stress. This was done at different time-points across the study (t_0_ – t_8_). All participants were asked after t_0_, t_1_, and t_2_, for the other time-points state anxiety was assessed for a subset of the sample. State anxiety across all time-points was measured for 62 participants.* | Laux, Lothar; Glanzmann, P.; Schaffner, P., &. Spielberger, C. D. (1981): *Das State-Trait-Angstinventar (STAI): theoretische Grundlagen und Handanweisung*, Weinheim: Beltz. |

**Post-experimental questionnaire**

***Questions regarding the experiment***

| **Item** | **Response options** |
| --- | --- |
| How interesting was the study for you? | Visual analogue scale from 0 “not interesting at all” to 100 “very interesting” |
| Were the instructions clear? | Visual analogue scale from 0 “very difficult to understand” to 100 “very easy to understand” |
| If not, what exactly was unclear? | open response |

***Experience with the olfactometer***

The participants rated their experience with the olfactometer on five questions using visual analogue scales (α_cronbach_ = .79).

| **Item** | **Response options** |
| --- | --- |
| Have you experienced the nasal pieces as annoying during the preparation? | Visual analogue scale from 0 “not at all annoying” to 100 “very annoying” |
| Have you experienced the nasal pieces as annoying while talking? | Visual analogue scale from 0 “not at all annoying” to 100 “very annoying” |
| Overall, did you find the nasal pieces rather pleasant or rather unpleasant? | Visual analogue scale from 0 “rather unpleasant” to 100 “rather pleasant” |
| How strongly did you notice the nasal pieces overall during the experiment? | Visual analogue scale from 0 “not at all” to 100 “very strongly” |
| Did the nasal pieces make you feel stressed during the interview? | Visual analogue scale from 0 “not at all” to 100 “very much” |

***Perception of the odor***

The present odor was rated with two items using visual analogue scales and one questions with an open response field.

| **Item** | **Response options** |
| --- | --- |
| How pleasant did you find the odor presented via the nasal pieces? | Visual analogue scale from 0 “very unpleasant” to 100 “very pleasant” (midpoint: neutral) |
| How intense did you find the odor presented via the nasal pieces? | Visual analogue scale from 0 “not perceptible” to 100 “very intense” |
| Were you able to recognize the odor? If so, what did it remind you of? | open response |

***Questions about sports***

| **Item** | **Response options** |
| --- | --- |
| How often per week do you normally exercise? | - daily - several times a week - once a week - less than once a week - not at all |
| How long do you train for (in minutes)? | open response |
| What sports do you do? | open response |
| Do you participate in competitions? | - yes - no |

***Questions about the rules of conduct and acute symptoms that might affect olfaction***

| **Item** | **Response options** |
| --- | --- |
| Have you eaten garlic in the last 24 hours? | - yes - no |
| Have you eaten onions in the last 24 hours? | - yes - no |
| Have you eaten any of the following foods in the last 24 hours? (please tick as appropriate) | - spicy dishes - asparagus - herbs - yogurt - lamb - strong smelling cheese - cabbage - celery - none of the above |
| Have you smoked in the last 24 hours? | - yes - no |
| If yes: How many cigarettes have you smoked in the last 24 hours? | open response (numeric) |
| Have you smoked in the past 7 days? | - yes - no |
| If yes: How many cigarettes have you smoked in the last 7 days? | open response (numeric) |
| Did you drink any alcohol within the past 24 hours | - yes - no |
| If yes: What and how much did you drink? | open response |
| Have you used any controlled substances or recreational drugs in the last four weeks? | - yes - no |
| If yes:   - What drugs have you used? - How many days ago did you use drugs? | open response fields |
| Have you taken any medication in the last 24 hours? | - yes - no |
| If yes:   - What medication have you taken? - In what dose did you take these medications? | open response fields |
| Have you used deodorant today? | - yes - no |
| How many hours did you sleep last night? | selection between 0 and 24 hours |
| What time did you get up this morning? | selection between 04:00 am and 5:00 pm |
| Since we are investigating the effect of the hormone cortisol, the following question is also important:  Have you been sexually active in the last 24 hours? | - yes - no |
| If yes: I was sexually active... | - alone - with another person |
| Do you live in an apartment with your partner? | - yes - no |
| Do you sleep in the same bed as your partner? | - yes - no |
| How often do you sleep in the same bed as your partner? (Please indicate how often per week or month) | Two open response fields for “per week” and “per moth” |
| Do you touch each other while sleeping? | - yes - no |
| How often do you have sex on average? | - daily - several times a week - once a week - several times a month - once a month - less than once a month - never |
| Has anything out of the ordinary happened today? | open response |
| How many hours do you work per week? | open response (numeric) |
| Do you have hay fever? | - yes - no |
| If yes: Do you experience acute symptoms of hay fever? | - yes - no |
| Do you currently have any other problems that could affect your ability to smell? | - yes - no |
| If yes: Which? | open response field |
| Are you currently using a nasal spray or other medication or ointments for the respiratory tract? | - yes - no |
| If yes: Which? | open response field |
| Do you have a cold today? | - yes - no |
| Have you had a cold in the last 7 days? | - yes - no |

***Importance of odors***

A single item was used to assess participants’ self-rated smelling ability and seven items were used to assess the general perception of body odors on visual analogue scales (α_cronbach_ = .70).

| **Item** | **Response options** |
| --- | --- |
| How would you rate your sense of smell? | Visual analogue scale from 0 “bad” to 100 “excellent” |
| Is it important to you how other people smell? | Visual analogue scale from 0 “unimportant” to 100 “very important” |
| How often do you notice other people's body odor in everyday life and find it unpleasant? | Visual analogue scale from 0 “very rare” to 100 “very often” |
| How often do you notice other people's body odor in everyday life and find it pleasant? | Visual analogue scale from 0 “very rare” to 100 “very often” |
| How well can you remember people's odors? | Visual analogue scale from 0 “not at all” to 100 “very good” |
| Do you think you could recognize people close to you by their odor? | Visual analogue scale from 0 “not at all” to 100 “definitely” |
| Are you consciously aware of your own body odor? | Visual analogue scale from 0 “not at all” to 100 “definitely” |
| Does the odor of people around you (e.g., when shopping, on the streetcar, in the waiting room) affect your mood? | Visual analogue scale from 0 “not at all” to 100 “definitely” |

***Body odor of the partner***

Five items were used to measure participants’ general associations with their partner’s odor with a visual analogue scale (α_cronbach_ = .82).

| **Item** | **Response options** |
| --- | --- |
| I love the smell of my partner. | Visual analogue scale from 0 “not true at all” to 100 “absolutely true” |
| I find the smell of my partner sexy. | Visual analogue scale from 0 “not true at all” to 100 “absolutely true” |
| The smell of my partner calms me down. | Visual analogue scale from 0 “not true at all” to 100 “absolutely true” |
| It is important to me how my partner smells. | Visual analogue scale from 0 “not true at all” to 100 “absolutely true” |
| The smell of my partner influences my mood. | Visual analogue scale from 0 “not true at all” to 100 “absolutely true” |

***Adapted questionnaires***

| *German version of the trait questionnaire of the State Trait Anxiety Inventory (STAI)* ^1^ | Laux, Lothar; Glanzmann, P.; Schaffner, P., &. Spielberger, C. D. (1981): *Das State-Trait-Angstinventar (STAI): theoretische Grundlagen und Handanweisung*, Weinheim: Beltz. |
| --- | --- |
| *Women* *were asked a German DSM-IV-TR-based questionnaire for the screening of premenstrual symptoms* ^9^ | Ditzen, B., Nussbeck, F., Drobnjak, S., Spörri, C., Wüest, D., & Ehlert, U. (2011). Validierung eines deutschsprachigen DSM-IV-TR basierten Fragebogens zum prämenstruellen Syndrom. *Zeitschrift für Klinische Psychologie und Psychotherapie*, *40*(3), 149–159. https://doi.org/10.1026/1616-3443/a000095 |
| *All participants were presented with a questionnaire measuring the individual significance of olfaction* ^10^ | Croy, I., Buschhüter, D., Seo, H. S., Negoias, S., & Hummel, T. (2010). Individual significance of olfaction: development of a questionnaire. *European Archives of Oto-Rhino-Laryngology*, *267*, 67–71. https://doi.org/10.1007/s00405-009-1054-0 |

***Olfactory Disorder***

| Do you have an impaired sense of smell (an olfactory disorder)? | - yes - no |
| --- | --- |
| If yes: Since when? | open response |

***Adapted questionnaires***

| *German version of the Perceived Stress Scale, (PSS-10)* ^2^ | Klein, E. M., Brähler, E., Dreier, M., Reinecke, L., Müller, K. W., Schmutzer, G., Wölfling, K., & Beutel, M. E. (2016). The German version of the Perceived Stress Scale – psychometric characteristics in a representative German community sample. *BMC Psychiatry*, *16*(1), 159. https://doi.org/10.1186/s12888-016-0875-9 |
| --- | --- |
| *Trier Inventory on chronic stress (TICS)* ^3^ | Schulz, P., & Schlotz, W. (1999). Trierer Inventar zur Erfassung von chronischem Streß (TICS): Skalenkonstruktion, teststatistische Überprüfung und Validierung der Skala Arbeitsüberlastung. *Diagnostica*, *45*(1), 8–19. https://doi.org/10.1026//0012-1924.45.1.8 |

Finally, participants where offered an open response field to add any comments they had about the study, before being thoroughly debriefed, thanked, and paid.

| Is there anything else you would like to tell us? | open response |
| --- | --- |

**G: Composite Stress Measure Analyses**

Similar to previous studies using the TSST ^11,12^, participants were asked to indicate to what degree they experienced physical discomfort, the desire to leave the situation, the desire for support in the situation, feelings of control (reverse coded). As in previous studies (Berger et al., 2016), the control item was only weakly correlated with the other items and was thus omitted from a combined scale. The internal consistency for the remaining four items (including the explicit stress item) ranged from αcronbach = .74 to αcronbach = .90 and the mean score was used as an alternative composite measure of overall stress responsiveness.

The same predictors were used as for the single item perceived stress measure. For a summary of the model see Table G1. Significant main effects were observed for sex (indicating higher stress levels for women) and time segment 2. The interactions between the TSST condition and odor as well as between the TSST conditions and each respective time segment were also significant. None of the other effects reached significance. For a depiction of these results see Figure G1. The interaction pattern indicates, that stress assessed via the composite stress measure increased in response to the TSST in the TSST-stress condition up to t_2_ and decreased from then on. In addition, in the partner odor condition higher stress levels were reported in the TSST-stress condition but not in the TSST-control condition. This difference remained stable across all times of measurement.

**Table G1**

Mixed linear effects model predicting the composite stress measure.

| *Predictors* | *b* | *SE(b)* | *CI* | *t* | *df* | *p* |
| --- | --- | --- | --- | --- | --- | --- |
| Intercept | 24.09 | 2.94 | 18.31 – 29.88 | 8.20 | 276.94 | <.001 |
| TSST condition¹ | -4.66 | 3.83 | -12.18 – 2.87 | -1.22 | 306.36 | .225 |
| Odor condition² | -4.76 | 3.87 | -12.37 – 2.85 | -1.23 | 306.56 | .219 |
| Time segment 1³ | -1.43 | 0.96 | -3.30 – 0.45 | -1.49 | 1412.10 | .136 |
| Time segment 2⁴ | 0.88 | 0.30 | 0.30 – 1.47 | 2.98 | 1412.35 | .003 |
| Sex⁵ | 5.46 | 2.37 | 0.77 – 10.15 | 2.30 | 174.08 | .023 |
| TSST condition¹ × Odor² | 11.53 | 5.47 | 0.76 – 22.29 | 2.11 | 305.72 | .036 |
| Time segment 1³ × TSST condition¹ | 5.09 | 1.36 | 2.42 – 7.76 | 3.74 | 1412.16 | <.001 |
| Time segment 1³ × Odor² | 2.55 | 1.37 | -0.14 – 5.25 | 1.86 | 1412.09 | .064 |
| Time segment 2⁴ × TSST condition¹ | -2.18 | 0.42 | -3.01 – -1.35 | -5.14 | 1412.50 | <.001 |
| Time segment 2⁴ × Odor² | -0.21 | 0.43 | -1.05 – 0.63 | -0.49 | 1412.29 | .624 |
| Time segment 1³ × TSST condition¹ × Odor² | -0.12 | 1.94 | -3.92 – 3.68 | -0.06 | 1412.12 | .951 |
| Time segment 2⁴ × TSST condition¹ × Odor² | -0.45 | 0.60 | -1.63 – 0.73 | -0.75 | 1412.38 | .455 |
| **Random Effects** | | | | | | |
| σ^2^ | 118.72 | | | | | |
| τ_00_ _ID_ | 235.58 | | | | | |
| ICC | .66 | | | | | |
| Observations (*N*_ID_ = 179) | | 1599 | | | | |
| Marginal R^2^ / Conditional R^2^ | | .082 / .692 | | | | |

*Note.* ¹ control = 0, stress = 1, ² control = 0, partner = 1, ³ breakpoint at t_2_: t_0_ = 0, t_1_ = 1, t_2_ to t_8_ = 2, ⁴ breakpoint at t_2_: t_0_ to t_2_ = 0, t_3_ = 1, t_4_ = 2, t_5_ = 3, t_6_ = 4, t_7_ = 5, t_8_ = 6, ⁵ male = 0, female = 1.

**Figure G1**

Composite stress measure by experimental conditions.

*Note.* Transparent dots represent raw data points. Solid points show mean values for each measurement. Error bars show 95%-confidence intervals of the mean based on random intercepts at the participant level. Violins visualize distributions. Solid lines show the prediction of the mixed model with 95% confidence intervals as shaded areas.

**H: Participant Flow** **
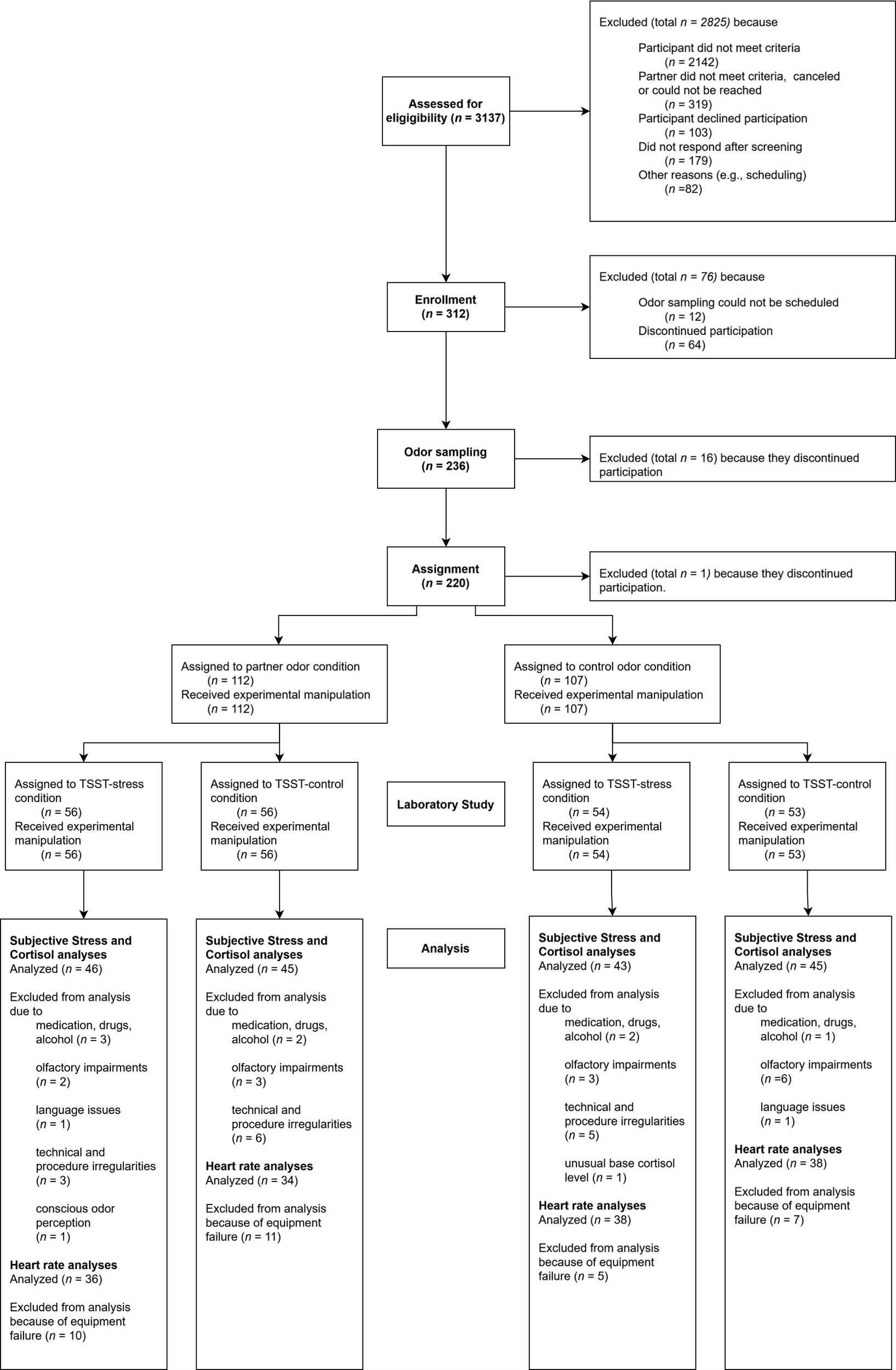
**

**References**

1. Laux, L., Glanzmann, P., Schaffner, P. & Spielberger, C. D. *Das State-Trait-Angstinventar*. (Göttingen, 1981).

2. Klein, E. M. *et al.* The German version of the Perceived Stress Scale – psychometric characteristics in a representative German community sample. *BMC Psychiatry* **16**, 159 (2016).

3. Schulz, P. & Schlotz, W. Trierer Inventar zur Erfassung von chronischem Streß (TICS): Skalenkonstruktion, teststatistische Überprüfung und Validierung der Skala Arbeitsüberlastung. *Diagnostica* **45**, 8–19 (1999).

4. Kelly, M. M., Tyrka, A. R., Anderson, G. M., Price, L. H. & Carpenter, L. L. Sex differences in emotional and physiological responses to the Trier Social Stress Test. *Journal of Behavior Therapy and Experimental Psychiatry* **39**, 87–98 (2008).

5. Yarnoz, M. J. & Curtis, A. B. More Reasons Why Men and Women Are Not the Same (Gender Differences in Electrophysiology and Arrhythmias). *The American Journal of Cardiology* **101**, 1291–1296 (2008).

6. Liu, J. J. W. *et al.* Sex differences in salivary cortisol reactivity to the Trier Social Stress Test (TSST): A meta-analysis. *Psychoneuroendocrinology* **82**, 26–37 (2017).

7. Finke, J. B., Hahn, S., Schächinger, H. & Klucken, T. Increased pupil and heart-rate responses to sexual stimuli in men after physical exertion. *Psychophysiology* **60**, e14254 (2023).

8. ter Kuile, M. M., Vigeveno, D. & Laan, E. Preliminary evidence that acute and chronic daily psychological stress affect sexual arousal in sexually functional women. *Behaviour Research and Therapy* **45**, 2078–2089 (2007).

9. Ditzen, B. *et al.* Validierung eines deutschsprachigen DSM-IV-TR basierten fragebogens zum prämenstruellen SyndromValidierung eines deutschsprachigen DSM-IV-TR basierten fragebogens zum prämenstruellen syndrom. 149–159 (2011).

10. Croy, I., Buschhüter, D., Seo, H.-S., Negoias, S. & Hummel, T. Individual significance of olfaction: development of a questionnaire. *European Archives of Oto-Rhino-Laryngology* **267**, 67–71 (2010).

11. Berger, J., Heinrichs, M., von Dawans, B., Way, B. M. & Chen, F. S. Cortisol modulates men’s affiliative responses to acute social stress. *Psychoneuroendocrinology* **63**, 1–9 (2016).

12. Hofer, M. K., Collins, H. K., Whillans, A. V. & Chen, F. S. Olfactory cues from romantic partners and strangers influence women’s responses to stress. *Journal of Personality and Social Psychology* **114**, 1–9 (2018).
